# Supplementary material for: Determinants of chemoselectivity in ubiquitination by the J2 family of ubiquitin-conjugating enzymes
Source: EMBO J. 2024 Nov 12;43(24):6705–39. doi: 10.1038/s44318-024-00301-3 (PMC11649903; doi:10.1038/s44318-024-00301-3)
Supplement: Supplementary file 1 — Appendix [file 44318_2024_301_MOESM1_ESM.pdf]

Appendix

Determinants of chemoselectivity in ubiquitination by the J2 family of ubiquitin conjugating enzymes

Anuruti Swarnkar[1], Florian Leidner[2], Ashok K. Rout [3,5], Sofia Ainatzi[4], Claudia Schmidt[1,6], Stefan Becker[3], Henning Urlaub[4], Christian Griesinger[3], Helmut Grubmüller[2], Alexander Stein[1]

Affiliations:

1 Research Group Membrane Protein Biochemistry, Max Planck Institute for Multidisciplinary Sciences, Am Fassberg 11, D-37077 Göttingen, Germany

2 Department of Theoretical and Computational Biophysics, Max Planck Institute for Multidisciplinary Sciences, Am Fassberg 11, D-37077 Göttingen, Germany

3 Department of NMR-based Structural Biology, Max Planck Institute for Multidisciplinary Sciences, Am Fassberg 11, D-37077 Göttingen, Germany

4 Research Group Bioanalytical Mass Spectrometry, Max Planck Institute for Multidisciplinary Sciences, Am Fassberg 11, D-37077 Göttingen, Germany

5 Current Address: Institut für Chemie und Metabolomics, Universität zu Lübeck, 23562 Lübeck

6 Current Address: ETH Zürich, Otto-Stern-Weg 3, 8093 Zürich, Switzerland

Correspondence to: alexander.stein@mpinat.mpg.de

Table of Contents

Appendix Figure S1 – Multiple sequence alignment of all human E2s 2

Appendix Figure S2 – Phylogenetic tree of Ube2J1 and Ube2J2 homologs 3

Appendix Figure S3 – Conserved elements in the J1 family 4

Appendix Figure S4 – Technical characterization of proteoliposomes 5

Appendix Figure S5 – Discharge of Ubc6-Ub by small nucleophile 6

Appendix Figure S6 – RING-meditated enhancement of Ubc6 and Ube2J2 activity 8

Appendix Table S1 – Data collection and refinement statistics for Ubc6 structures 10

Appendix Table S2 – Data collection and refinement statistics for Ubc6-Ub structure 11

Appendix References 12

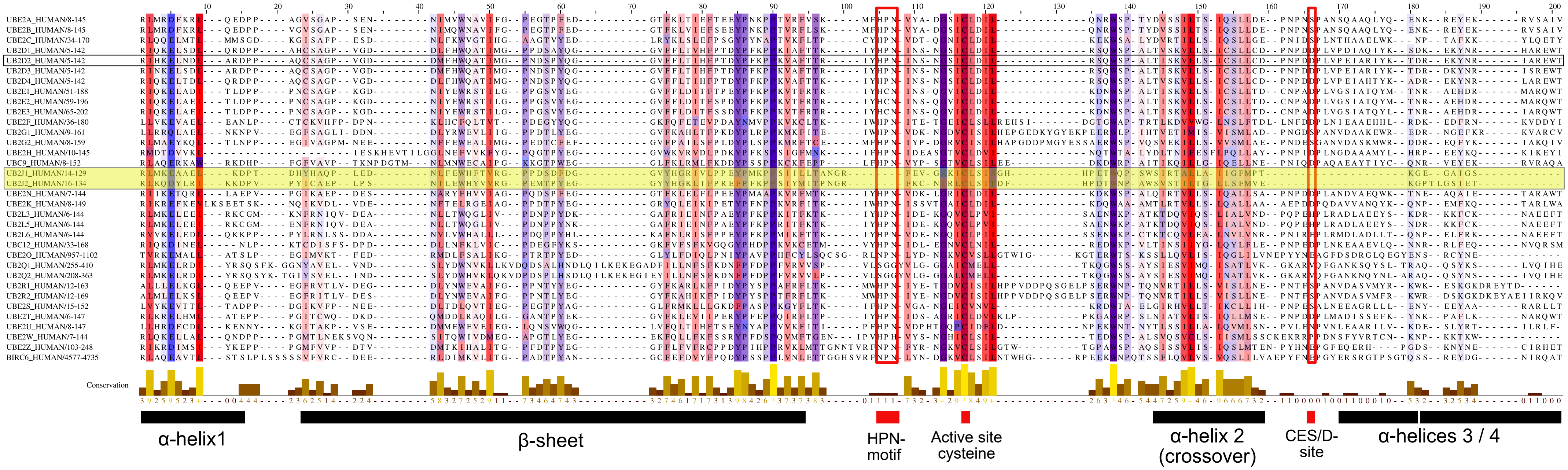

Appendix Figure S1 - Multiple sequence alignment of human E2s

Multiple sequence alignment (MSA) of the UBC domains of 34 human ubiquitin conjugating enzymes. The alignment was generated using the MAFFT L-INS-I algorithm and visualized in Jalview with coloring according to hydrophobicity and conservation. Secondary structure elements of the UBC domain, and functionally important residues such as the active site Cys, the HPN motif and the CES/D site are annotated. Bars and numbering below the alignment indicate sequence conservation. For orientation, a canonical E2, Ube2D2, and the two human J subfamily members Ube2J1 and Ube2J2 are highlighted.

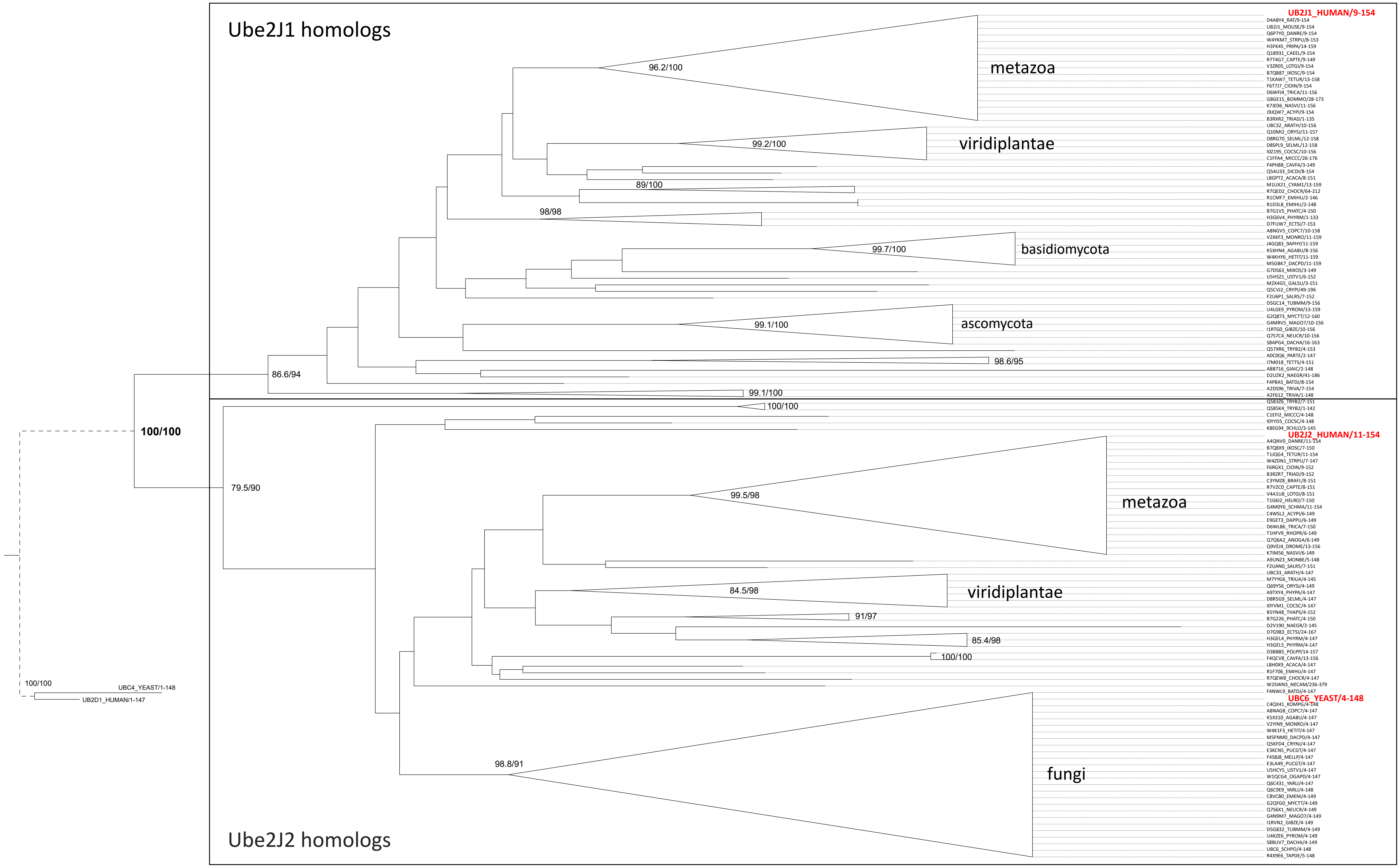

Appendix Figure S2 - Phylogenetic tree of Ube2J1 and Ube2J2 homologs - *Figure legend on the next page*

Appendix Figure S2 - Phylogenetic tree of Ube2J1 and Ube2J2 homologs *(shown on the previous page)*

A multiple sequence alignment (MSA) of 126 Ube2J1 and Ube2J2 homologs was generated using MAFFT (Katoh & Standley, 2013). Sequences of yeast Ubc4 and human Ube2D1 were included as outgroup. A phylogenetic tree was calculated with IQ-tree (Nguyen et al, 2015) and visualized using FigTree (http://tree.bio.ed.ac.uk/software/figtree/). The tree is unrooted, though the outgroup comprising Ubc4 and Ube2D1 is drawn at root. Numbers in parentheses are SH-aLRT support (%) / ultrafast bootstrap support (%) for well-supported branches, including the split into J1 and J2 subfamilies. Other branching patterns are poorly supported. For visualization some branches are collapsed. The MSA, and the non-collapsed tree can be accessed in Dataset EV2 and Dataset EV3, respectively.

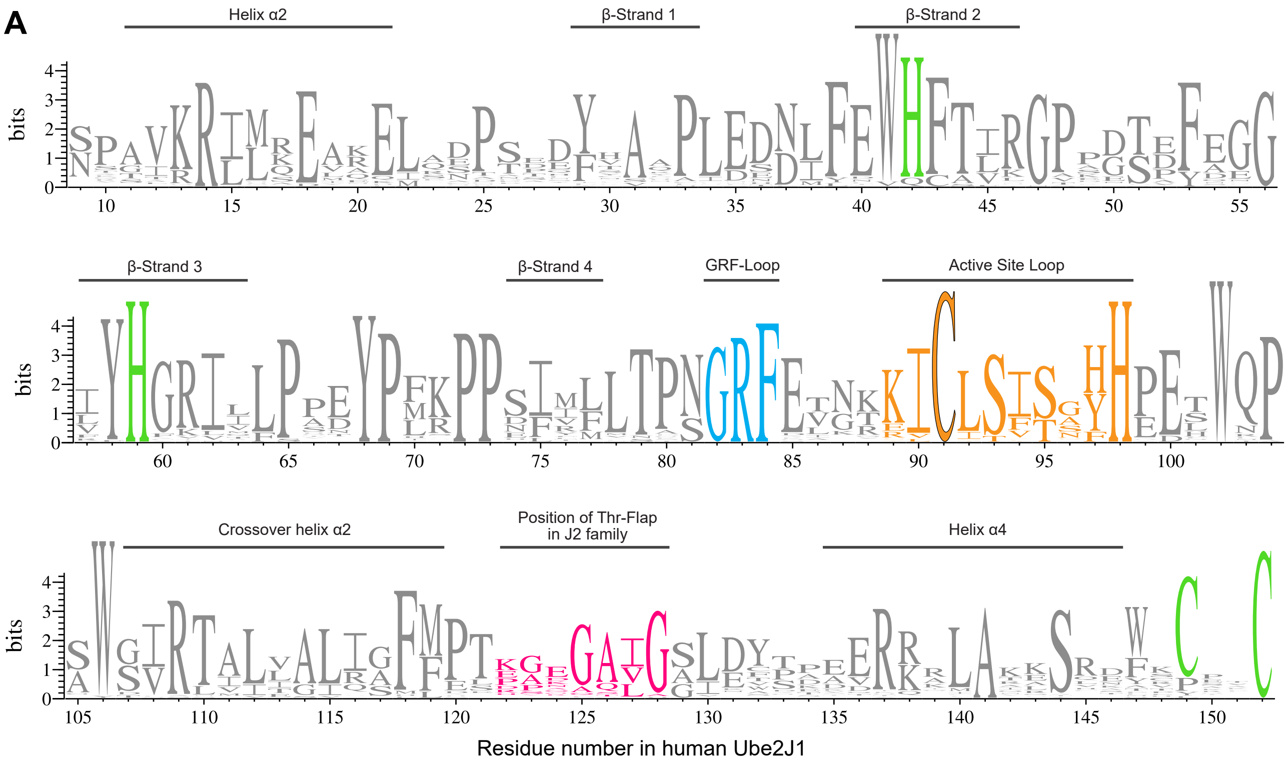

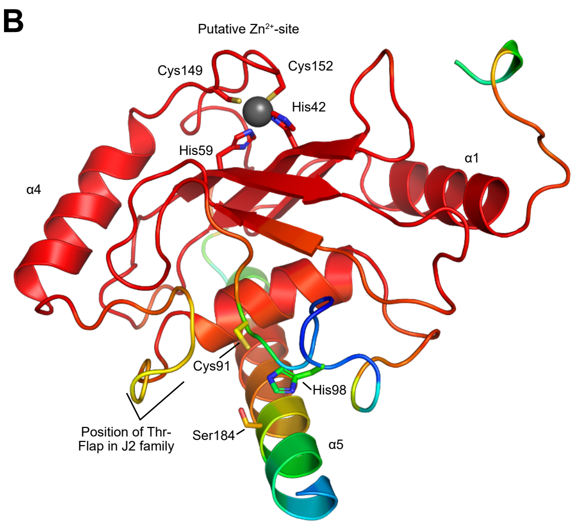

Appendix Figure S3 - Conserved elements in the J1 family

A Weblogo for the UBC domain of 54 Ube2J1 homologs from a diverse set of eukaryotes, selected based on the tree in Appendix Figure S2. The residue numbering corresponds to human Ube2J1. Conserved features are coloured as in Fig 1D, with the GRF motif in blue, the active site loop in orange. Notably, the Thr-flap is not conserved compared to the J2 family. The corresponding region in coloured in pink. Additionally, cysteine and histidine residues highly conserved in the J1, but not the J2 family, are shown in green. Variation in these sites in the J1 familiy occur predominantly in plants, as can be seen in the MSA used for generating the Weblogo (Dataset EV4).

B Cartoon representation of the structure of the UBC domain of Ube2J1 as predicted by Alphafold3 (Abramson et al, 2024). The full-length sequence of human Ube2J1 and a single Zn2+ ion were used as inputs. Only the UBC domain and an additional helix α5 are shown. Colouring according to the pLDDT per-atom confidence estimate. Some side chains are shown as sticks: The active site cysteine (Cys91), the catalytically important histidine (His98), the zink-chelating residues (His42, His59, Cys149, Cys152), and Ser184, which has been reported to get phosphorylated and to play a role in Ube2J1 regulation (Elangovan et al, 2017; Oh et al, 2006).

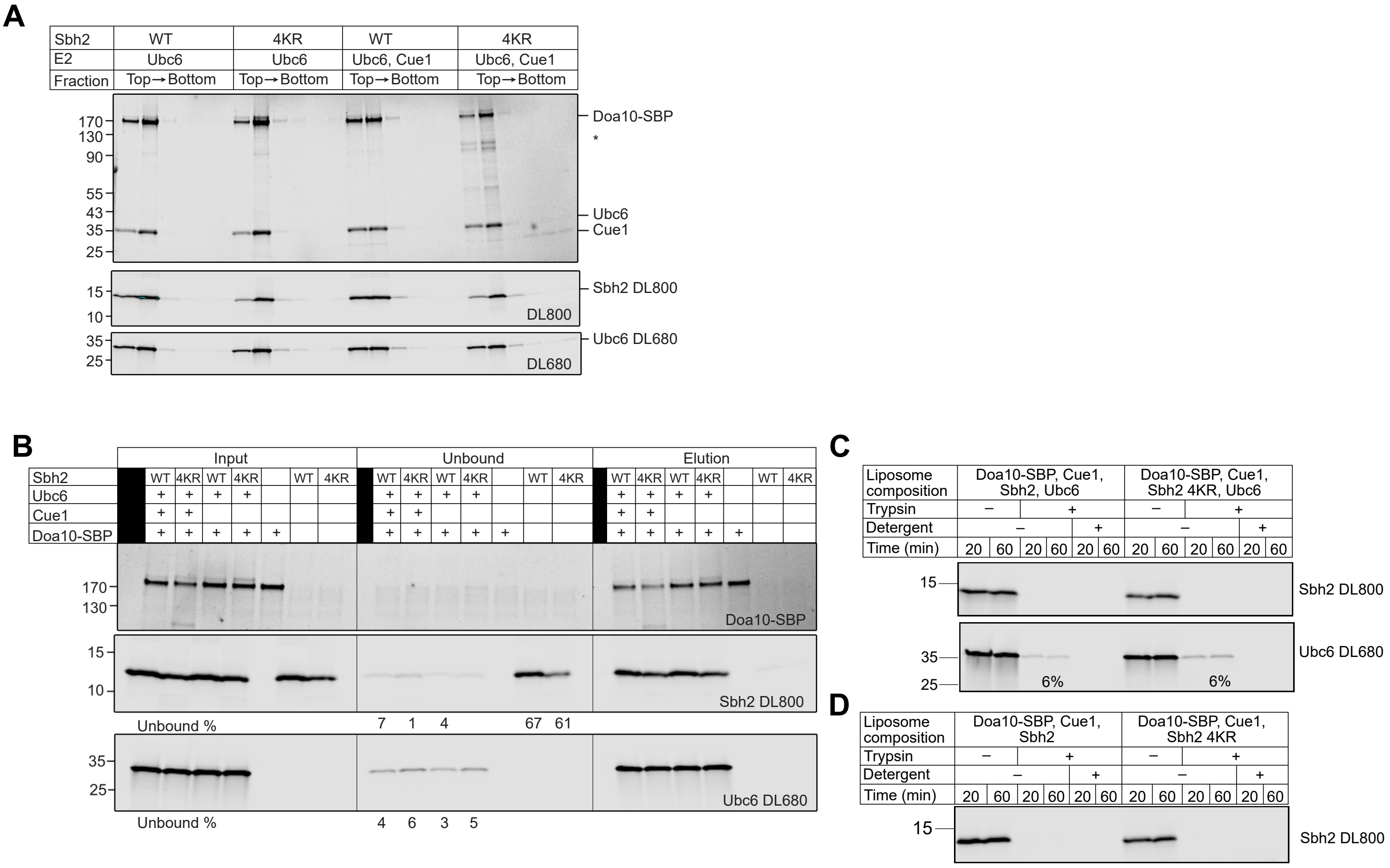

Appendix Figure S4 - Technical characterization of proteoliposomes, related to Fig 4

A Representative SDS-PAGE of a liposome floatation assay. Liposomes containing Doa10 and fluorescently labelled Ubc6 with or without Cue1 were co-reconstituted with fluorescently labelled WT Sbh2 and 4KR mutant. The co-reconstituted liposomes were floated in a Nycodenz step gradient. Gradient fractions were analysed by SDS-PAGE, followed by Coomassie staining (top) and fluorescence scanning (bottom). The asterisk indicates degradation products of Doa10.

B Representative SDS-PAGE of liposome pull-down assays to assess co-reconstitution. Liposomes containing Doa10-SBP, fluorescently labelled Ubc6 and either WT Sbh2 or Sbh2 4KR, co-reconstituted in the presence or absence of Cue1 were immobilized onto streptavidin magnetic beads via the C-terminal streptavidin binding peptide (SBP) tag on Doa10. Beads were then washed and eluted with biotin. Input, unbound and elution fractions were analysed by SDS-PAGE, followed by Coomassie staining (top) and fluorescence scanning (bottom). As a negative control for unspecific binding of liposomes to the bead material, liposomes containing only WT Sbh2 or Sbh2 4KR were included. Numbers below unbound fractions indicate the percentage of Sbh2 and Ubc6 relative to input. Low values indicate higher reconstitution efficiency.

C, D Representative SDS-PAGE of protease protection assays to assess orientation of Sbh2 and Ubc6 in the liposomal membrane. Liposomes containing Doa10, fluorescently labelled WT Sbh2 or 4KR and Cue1, co-reconstituted with (C) or without (D) Ubc6 were analysed. Liposomes were subjected to tryptic digest for the indicated time in the presence or absence of detergent (Triton X-100). Only correctly oriented proteins expose tryptic site on the liposomal surface. Low numbers thus indicate a high fraction of correctly oriented protein. Samples were analysed by fluorescence scanning.

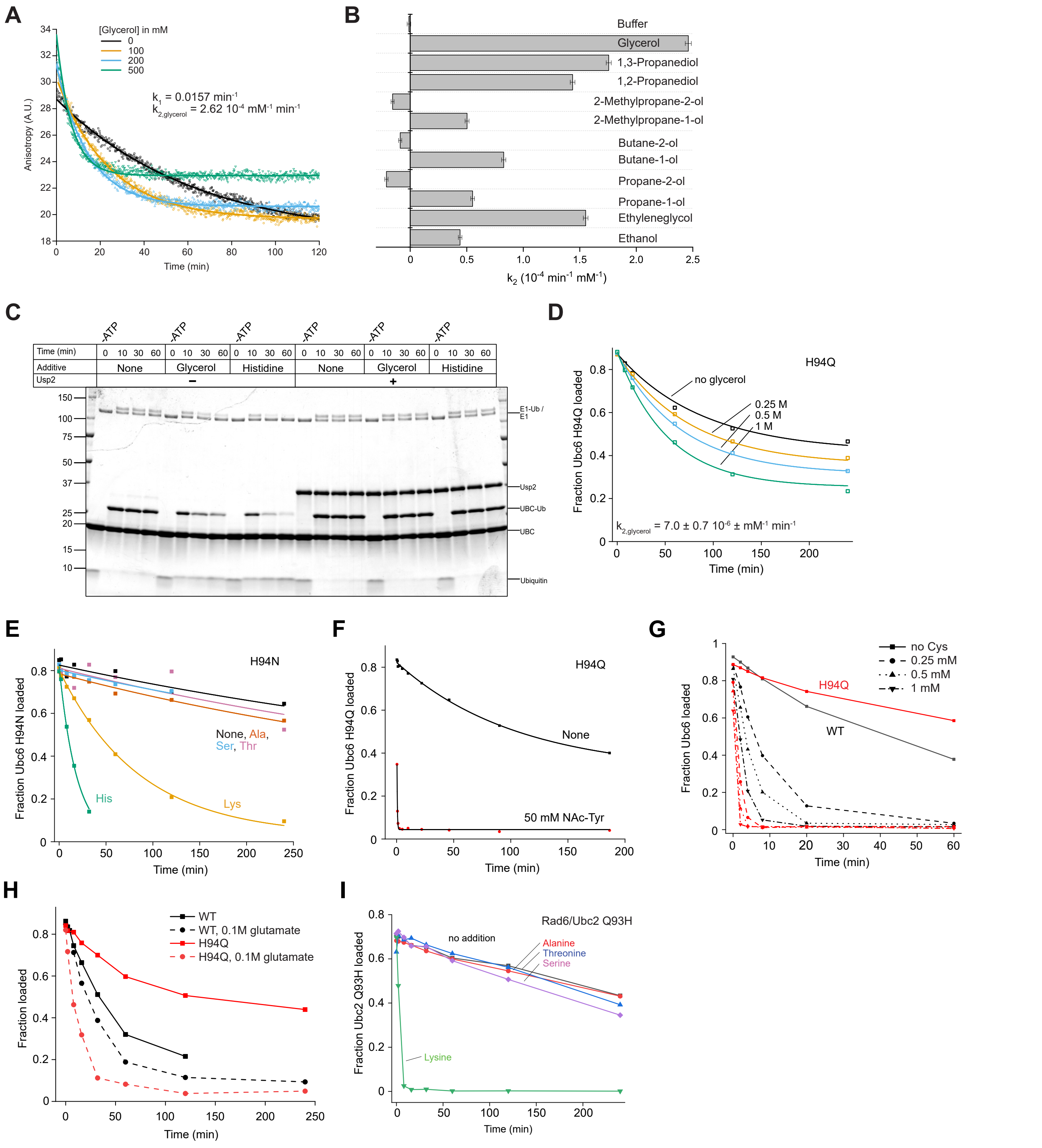

Appendix Figure S5 - Discharge of Ubc6-Ub by small nucleophile, related to Fig 5

A Ubc6 discharge assay measured by fluorescence anisotropy as described in Fig 5A. The Ubc6 UBC domain was loaded with Alexa Fluor 488 labelled ubiquitin by incubation with E1 and ATP. This reaction was diluted 1:10 into chase buffer containing EDTA and the indicated glycerol concentrations, followed by the start of the measurement with a delay of about 20 s. Anisotropy data were globally fitted with a mono-exponential function to obtain rate for hydrolysis (k_1) and discharge onto glycerol ($k_{2,glycerol}$).

B Bar plots of rate constants for Ubc6-Ub discharge by the indicated alcohol. Data was obtained and globally fitted as described in (A). Error bars denote the estimated error of the fit. Negative values suggest some denaturing effect of the particular alcohol.

continued on next page

Appendix Figure S5 - Discharge of Ubc6-Ub by small nucleophile, related to Fig 5 - *continued*

C Continuous E2 loading assay performed with 30 µM UBC domain of Ubc6, 10 µM ubiquitin, 0.5 µM E1, and 1 mM ATP, analyzed by non-reducing SDS PAGE and Coomassie staining. Continuous E1 activity ensures that ubiquitin discharged by hydrolysis is reloaded onto Ubc6. Therefore, free ubiquitin is depleted in reactions without an extra nucleophile and the fraction of Ub-loaded E1 and E2 is constant over the course of the experiment. In contrast, ubiquitin discharged by histidine or glycerol (each 100 mM) becomes inert because of a blocked C-terminus (see mass spectra in (C) and (I)), resulting in a gradual depletion of loaded E2 and E1, and the appearance of ubiquitin. Presence of the deubiquitinating enzyme Usp2 (5 µM) restores free ubiquitin and thus E2 loading.

D Ubiquitin discharge with the Ubc6 mutant H94Q in the presence of the indicated glycerol concentrations. Samples were analysed by non-reducing SDS-PAGE and stain-free imaging. The fraction of the loaded and discharged state were quantified by densitometry. The data was then globally fitted to determine rate constants for hydrolysis, and discharge by glycerol ($k_{2,glycerol}$), accounting for a minor fraction of autoubiquitination. Solid lines show the result of the fitting procedure. N=2; error denotes standard deviation.

E Ubiquitin discharge with the Ubc6 mutant H94N in the presence of 100 mM of the indicated free amino acids. Samples taken of the chase reaction were analysed by non-reducing SDS-PAGE and stain-free imaging. The fraction of the loaded and discharged state were quantified by densitometry. The data was then globally fitted to determine rate constants for hydrolysis, and discharge by the individual free amino acids. Solid lines show the result of the fitting procedure. N=1, k_1 = 0.0012 min^{-1}, $k_{2,Lys}$ = 1.0 10^{-4} mM^{-1} min^{-1}, $k_{2,His}$ = 5.2 10^{-4} mM^{-1} min^{-1}.

F-H Ubiquitin discharge with the Ubc6 mutant H94Q in the presence of the indicated concentrations of free amino acids. (F) NAc-Tyr, (G) cysteine, (H) glutamate. In (F), solid lines represent global fits. In (G) and (H) data for discharge of WT Ubc6 is shown for comparison and lines connect data points. In (G) data for WT Ubc6 is reproduced from Fig. EV4C.

I Ubiquitin discharge assay performed with Ub-loaded yeast Rad6/Ubc2 mutant Q93H, comparing discharge without and with 100 mM of the indicated free amino acids. Values for each time point are shown as coloured symbols connected by solid lines.

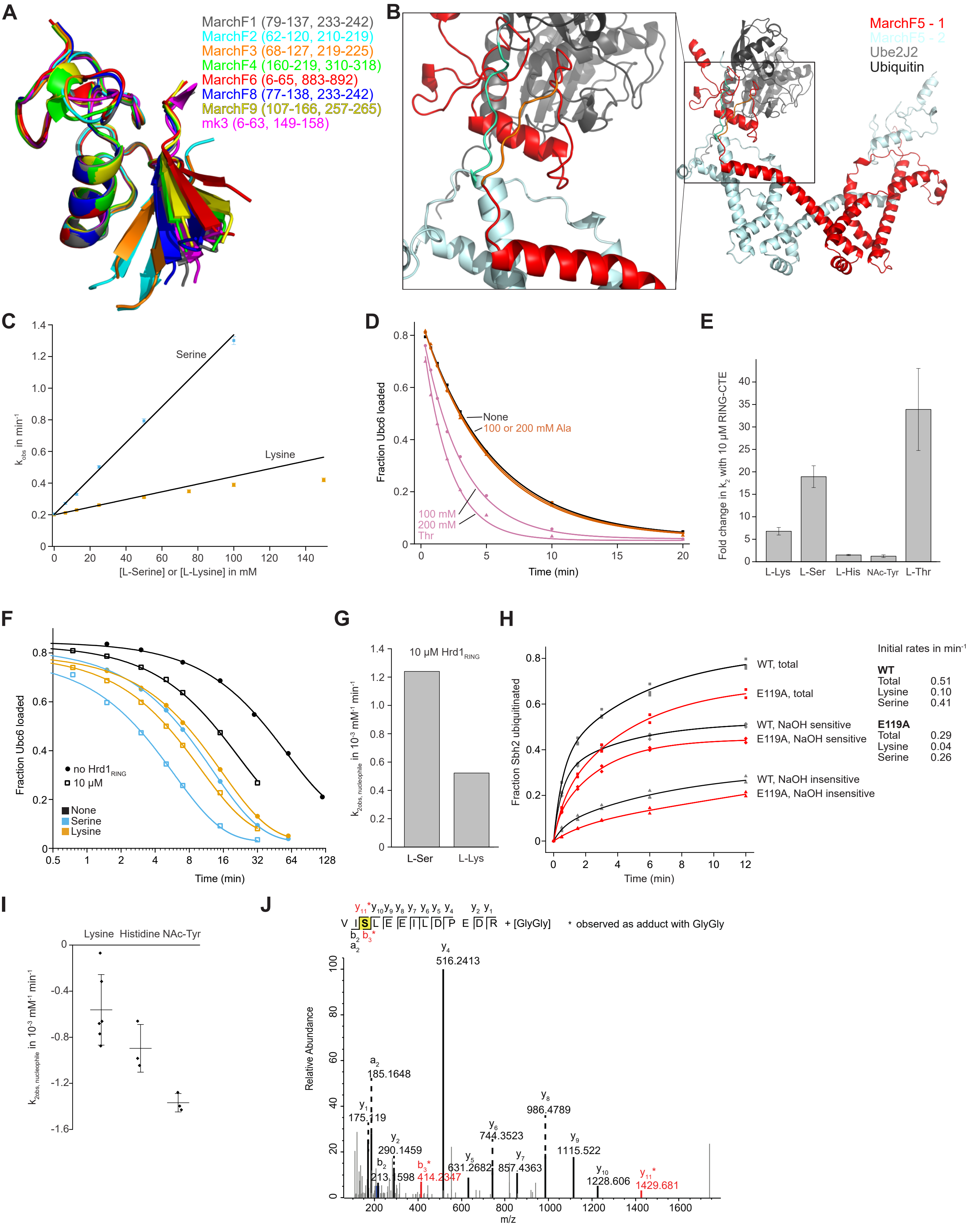

Appendix Figure S6 - **RING-meditated enhancement of Ubc6 and Ube2J2 activity** -
Figure legend on the next page

Appendix Figure S6 - RING-meditated enhancement of Ubc6 and Ube2J2 activity, related to Fig 6

A Overlay of Alphafold 2 predicted structures of the RING domains and C-terminal elements (CTE) of the indicated human MarchF E3 ligases and the viral mK3 ligase. Coordinates for March proteins were retrieved from Alphafold Protein Structure Database (Jumper et al, 2021). The structure of mk3 was predicted using ColabFold v1.5.5 (Mirdita et al, 2022) for mk3 from murid herpesvirus (MuHV-4, UniProt O41933). All structures were superimposed on the RING domain of MarchF6. Only the indicated regions are shown as cartoon representations.

B To predict the structure of dimeric human MarchF5 in complex with Ube2J2 and ubiquitin, ColabFold v1.5.5 (Mirdita et al, 2022) was used, fed with two copies of each protein. Predicted structures are shown in cartoon representation, but only one copy of Ube2J2 and ubiquitin is shown, in light and dark grey, respectively. The two copies of MarchF5 are shown in red and cyan. The zoomed-in image on the left shows the contact region between the N-terminal RING domain of the first copy of MarchF5 in orange, and the CTE of the second copy colored in aquamarine.

C To test, if free lysine or serine impair the interaction between Ubc6-Ub and RING-CTE, discharge reactions as in Fig EV5C were performed in the presence of 10 µM RING-CTE and the indicated concentrations of either free lysine or serine. From the fraction of loaded Ubc6, observed rate constants were derived, that did not distinguish between hydrolysis and discharge by the nucleophile. The obtained apparent rate constants were then plotted against the nucleophile concentration. Solid lines represent linear fits of the data between 0 and 50 mM nucleophile. Linearity for free serine indicates that serine does not impair RING-CTE binding. The deviation from linearity at high lysine concentrations suggests that free lysine interferes with this interaction. Therefore, 50 mM free lysine was used in experiment for Fig 6D and F.

D Ubiquitin discharge assays with WT Ubc6 performed as described in Fig EV5C but with indicated concentrations of free alanine or threonine in the presence of 10 µM RING-CTE. The data was globally fitted, with solid lines representing fit results. Fitting results are reported in Fig 6D, E.

E Bar plots comparing reactivity of Ubc6 towards the indicated nucleophiles in the absence or presence of 10 µM RING-CTE. Data from Fig 6D was used to calculate fold enhancement of discharge rates in the presence of 10 µM RING-CTE. Error bars were calculated based on the standard deviation for reactions in the absence or presence of RING-CTE. N (without/with RING-CTE) is (5/2) for l-Lys, (15/4) for l-Ser, l-His (5/2) for l-His, (3/3) for NAc-Tyr, and (3/2) for l-Thr.

F Ubiquitin discharge assays with WT Ubc6 performed in the presence of 50 mM of the indicated free amino acids and the indicated concentrations of the RING domain of yeast Hrd1. Data were globally fitted to mono-exponential functions to determine apparent rate constants for hydrolysis and discharge by free amino acids. Solid lines represent fit results.

G Bar plots comparing reactivity of Ubc6 towards the indicated nucleophiles in the presence of 10 µM Hrd1 RING from the experiment in (F).

H Time course of the emergence of NaOH-resistant and -sensitive Sbh2 ubiquitinations in reactions with co-reconstituted Doa10 and either wt Ubc6 or its mutant E119A. Samples collected at indicated time points were treated with NaOH to preserve only lysine modifications or were loaded untreated. Samples were analysed by SDS-PAGE followed by fluorescence scanning. The plot shows the quantification of the fraction of ubiquitinated Sbh2 from three experiments. Solid lines represent double exponential fits to the data. Fits were used to estimate initial rates reported in the adjacent table, where 'Serine' and 'Lysine' represent the NaOH-sensitive and -resistant fractions, respectively.

I Observed rate-constants for J2-Ub discharge in the presence of M6-RING-CTE and the indicated nucleophile. Reactions as shown in Fig EV5C were fitted as described, yielding negative values for reactions with lysine, histidine, and NAc-Tyr, indicating that these compounds interfere with E3 binding to Ube2J2.

J MS/MS spectrum of the indicated Ubc6 peptide modified with the di-glycine (GlyGly) ubiquitin remnant at Ser196.

Appendix Table S1 - Data collection and refinement statistics for Ubc6 structures

	**Ubc6 UBC_Cadmium PDB 9EWP**	**Ubc6 UBC_Citrate PDB 9EN5**
Wavelength (Å)	1	1
Resolution range (Å)	40.24 - 1.21 (1.253 - 1.21)	38.12 - 1.331 (1.378 – 1.331)
Space group	P 21 21 21	P 21 21 21
Unit cell parameters (Å, °)	*a* = 43.139, *b* = 44.169, *c* = 97.578<br>*α* = *β* = *γ* = 90	*a* = 44.02, *b* = 55.384, *c* = 76.211<br>*α* = *β* = *γ* = 90
Total reflections	709948 (59942)	496009 (18288)
Unique reflections	57616 (5615)	40931 (2752)
Multiplicity	12.3 (10.7)	12.1 (6.6)
Completeness (%)	99.55 (96.88)	94.15 (64.78)
Mean I/sigma (I)	25.47 (1.25)	31.10 (0.95)
Wilson B-factor ($Å^2$)	15.16	20.52
R-merge (%)	0.05455 (2.727)	0.03871 (1.39)
R-meas (%)	0.05684 (2.87)	0.04035 (1.508)
R-pim	0.0158 (0.8848)	0.01121 (0.5561)
CC1/2	0.999 (0.876)	1 (0.67)
CC*	1 (0.966)	1 (0.896)
Reflections used in refinement	57446 (5498)	40911 (2750)
Reflections used for R-free	2871 (273)	2041 (138)
R-work (%)	0.1357 (0.6310)	0.1609 (0.5874)
R-free (%)	0.1565 (0.6358)	0.1857 (0.5726)
CC (work)	0.971 (0.608)	0.968 (0.718)
CC (free)	0.965 (0.463)	0.968 (0.661)
Number of non-hydrogen atoms	1662	1650
macromolecules	1400	1387
ligands	39	0
solvent	241	263
Number of protein residues	172	169
RMSD (bonds) (Å)	0.019	0.005
RMSD (angles) (°)	1.61	0.87
Ramachandran favored (%)	98.24	98.20
Ramachandran allowed (%)	1.76	1.80
Ramachandran outliers (%)	0.00	0.00
Rotamer outliers (%)	0.00	0.00
Clashscore	2.13	3.62
Average B-factor ($Å^2$)	20.95	24.39
macromolecules	19.11	22.28
ligands	25.08	0
solvent	31.28	35.50

Values in parenthesis correspond to the highest resolution shell

Appendix Table S2 - Data collection and refinement statistics for the Ubc6-Ub structure

	**Ubc6 UBC isopetide linked Ub<br>PDB 9EYH**
Wavelength (Å)	1
Resolution range (Å)	48.01 - 2.6 (2.693 - 2.6)
Space group	I 1 2 1
Unit cell parameters (Å, °)	a = 105.753, b = 53.49, c = 111.798, α = 90, β = 103.152, γ = 90
Total reflections	230023 (22548)
Unique reflections	19017 (1903)
Multiplicity	12.1 (11.8)
Completeness (%)	99.67 (99.53)
Mean I/sigma (I)	17.41 (2.95)
Wilson B-factor (Å^2)	63.16
R-merge (%)	0.0848 (0.9275)
R-meas (%)	0.08864 (0.9696)
R-pim	0.02558 (0.2809)
CC1/2	0.999 (0.945)
CC*	1 (0.986)
Reflections used in refinement	18963 (18964)
Reflections used for R-free	895 (96)
R-work (%)	0.2343 (0.3392)
R-free (%)	0.2655 (0.3590)
CC (work)	0.944 (0.818)
CC (free)	0.937 (0.814)
Number of non-hydrogen atoms	3945
macromolecules	3945
ligands	0
solvent	0
Number of protein residues	489
RMSD, (bonds) (Å)	0.003
RMSD, (angles) (°)	0.52
Ramachandran favored (%)	97.30
Ramachandran allowed (%)	2.70
Ramachandran outliers (%)	0.00
Rotamer outliers (%)	0.90
Clashscore	7.09
Average B-factor (Å^2)	85.87
macromolecules	85.87
No. of TLS groups	15

Values in parenthesis correspond to the highest resolution shell

Appendix References

Abramson J, Adler J, Dunger J, Evans R, Green T, Pritzel A, Ronneberger O, Willmore L, Ballard AJ, Bambrick J *et al* (2024) Accurate structure prediction of biomolecular interactions with AlphaFold 3. *Nature* 630: 493-500

Elangovan M, Chong HK, Park JH, Yeo EJ, Yoo YJ (2017) The role of ubiquitin-conjugating enzyme Ube2j1 phosphorylation and its degradation by proteasome during endoplasmic stress recovery. *J Cell Commun Signal* 11: 265-273

Jumper J, Evans R, Pritzel A, Green T, Figurnov M, Ronneberger O, Tunyasuvunakool K, Bates R, Zidek A, Potapenko A *et al* (2021) Highly accurate protein structure prediction with AlphaFold. *Nature* 596: 583-589

Katoh K, Standley DM (2013) MAFFT multiple sequence alignment software version 7: improvements in performance and usability. *Mol Biol Evol* 30: 772-780

Mirdita M, Schütze K, Moriwaki Y, Heo L, Ovchinnikov S, Steinegger M (2022) ColabFold: making protein folding accessible to all. *Nature methods* 19: 679-+

Nguyen LT, Schmidt HA, von Haeseler A, Minh BQ (2015) IQ-TREE: a fast and effective stochastic algorithm for estimating maximum-likelihood phylogenies. *Mol Biol Evol* 32: 268-274

Oh RS, Bai X, Rommens JM (2006) Human homologs of Ubc6p ubiquitin-conjugating enzyme and phosphorylation of HsUbc6e in response to endoplasmic reticulum stress. *J Biol Chem* 281: 21480-21490
