# Supplementary figures and images for: Determinants of chemoselectivity in ubiquitination by the J2 family of ubiquitin-conjugating enzymes
Source: EMBO J. 2024 Nov 12;43(24):6705–39. doi: 10.1038/s44318-024-00301-3 (PMC11649903; doi:10.1038/s44318-024-00301-3)

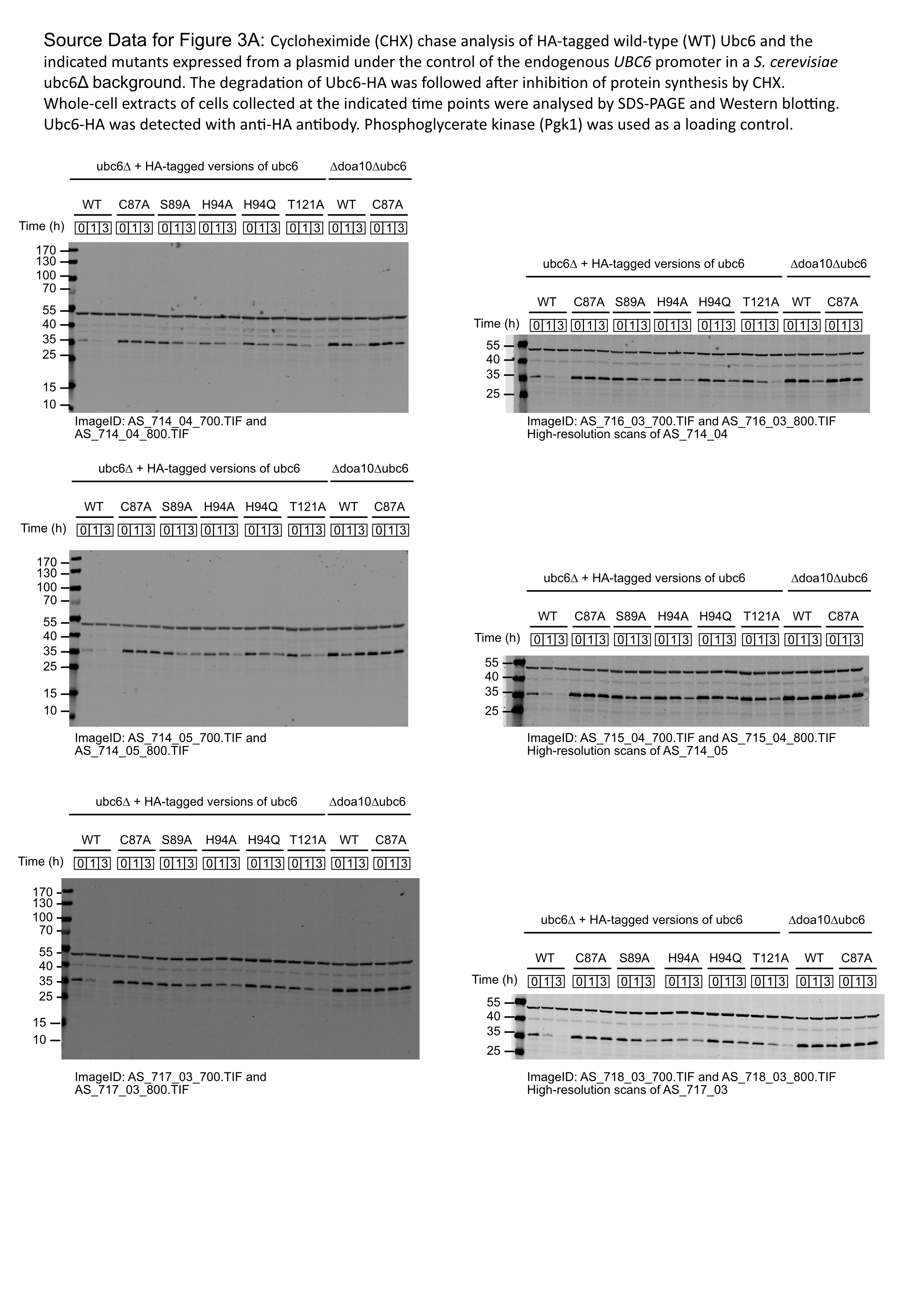

Supplement: Supplementary file 7 — Source data Fig. 3 [file 44318_2024_301_MOESM7_ESM.zip › SD figure 3/SD_Figure 3A.tiff]

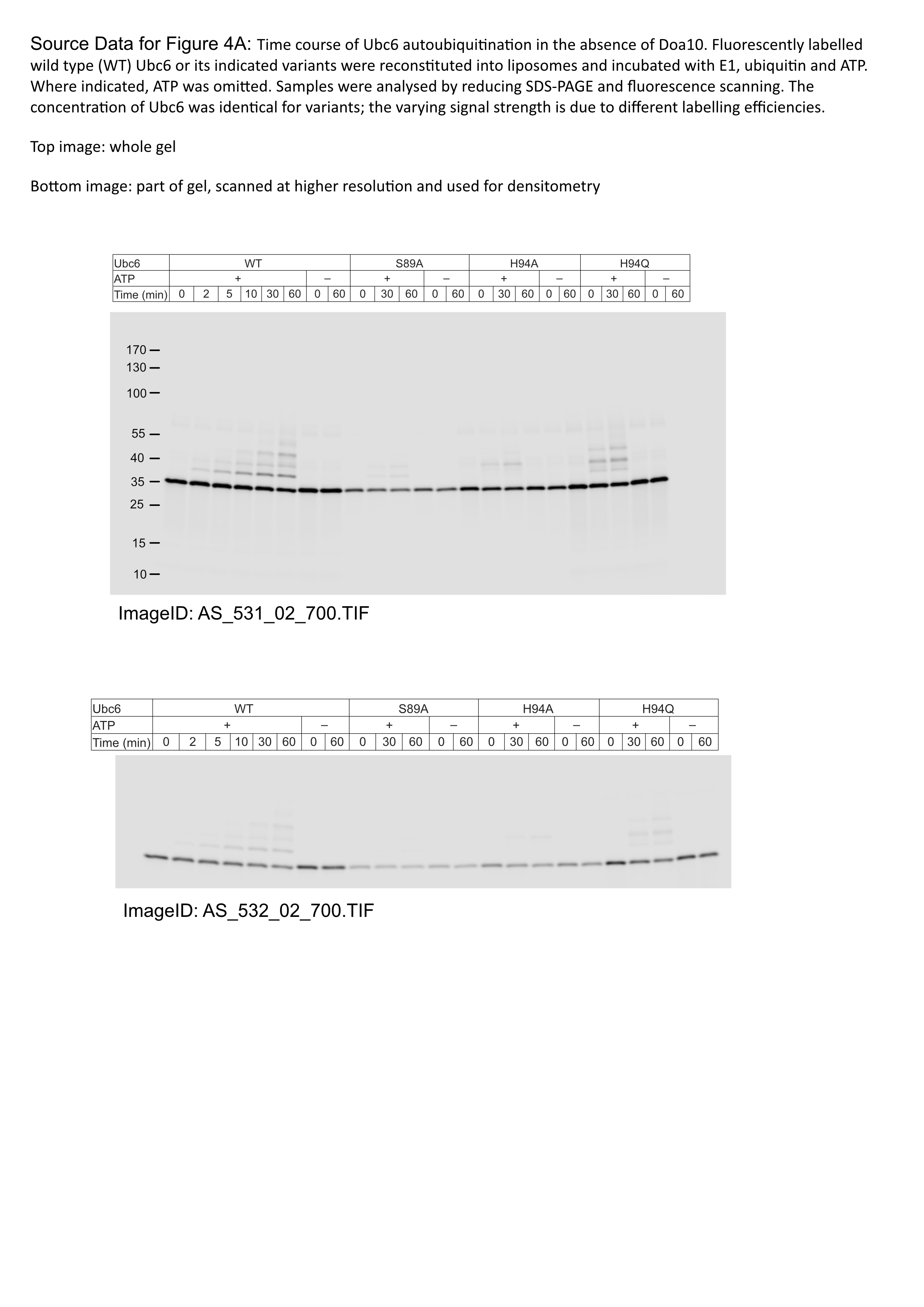

Supplement: Supplementary file 8 — Source data Fig. 4 [file 44318_2024_301_MOESM8_ESM.zip › SD figure 4/SD_Figure_4A.tiff]

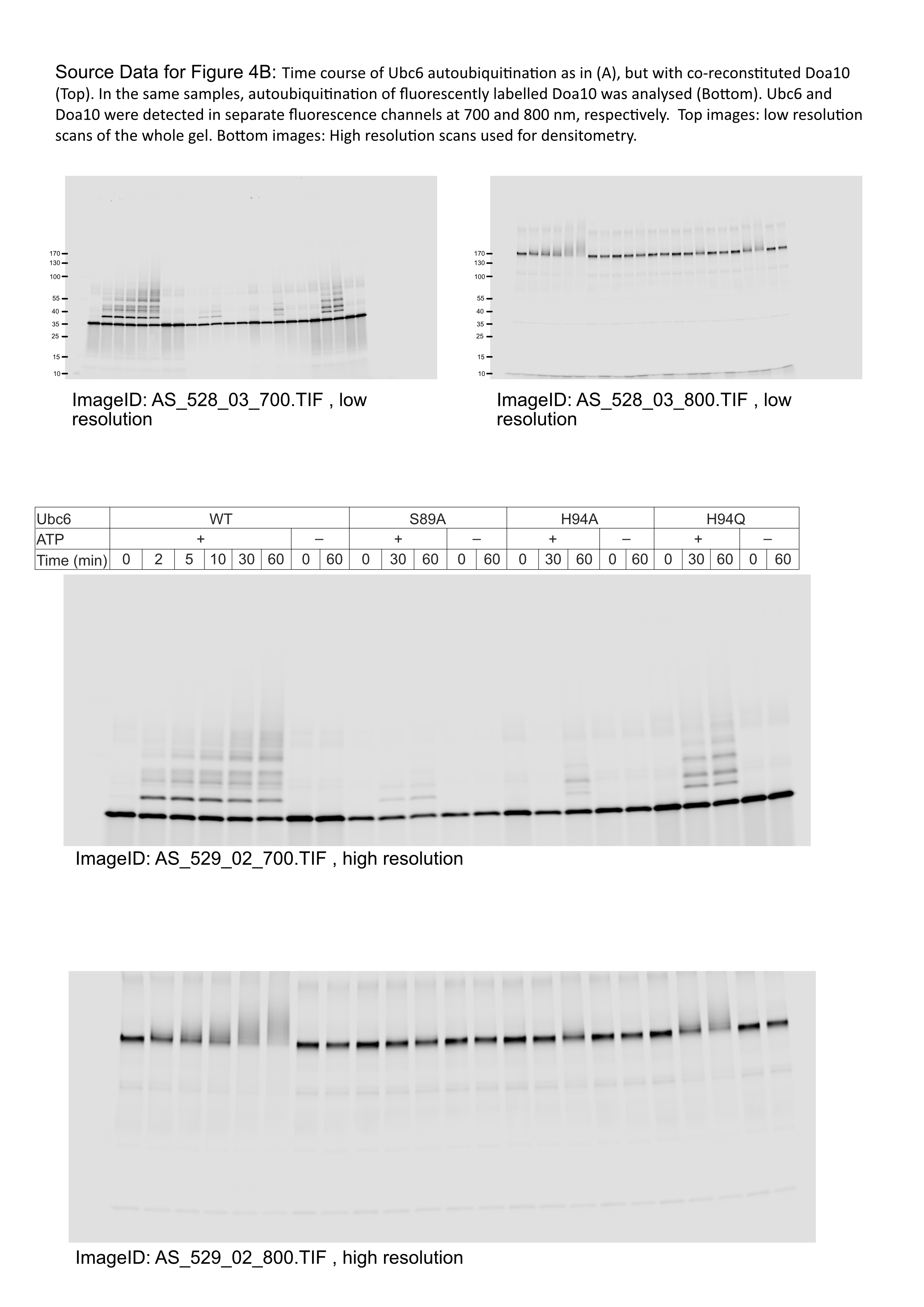

Supplement: Supplementary file 8 — Source data Fig. 4 [file 44318_2024_301_MOESM8_ESM.zip › SD figure 4/SD_Figure_4B.tiff]

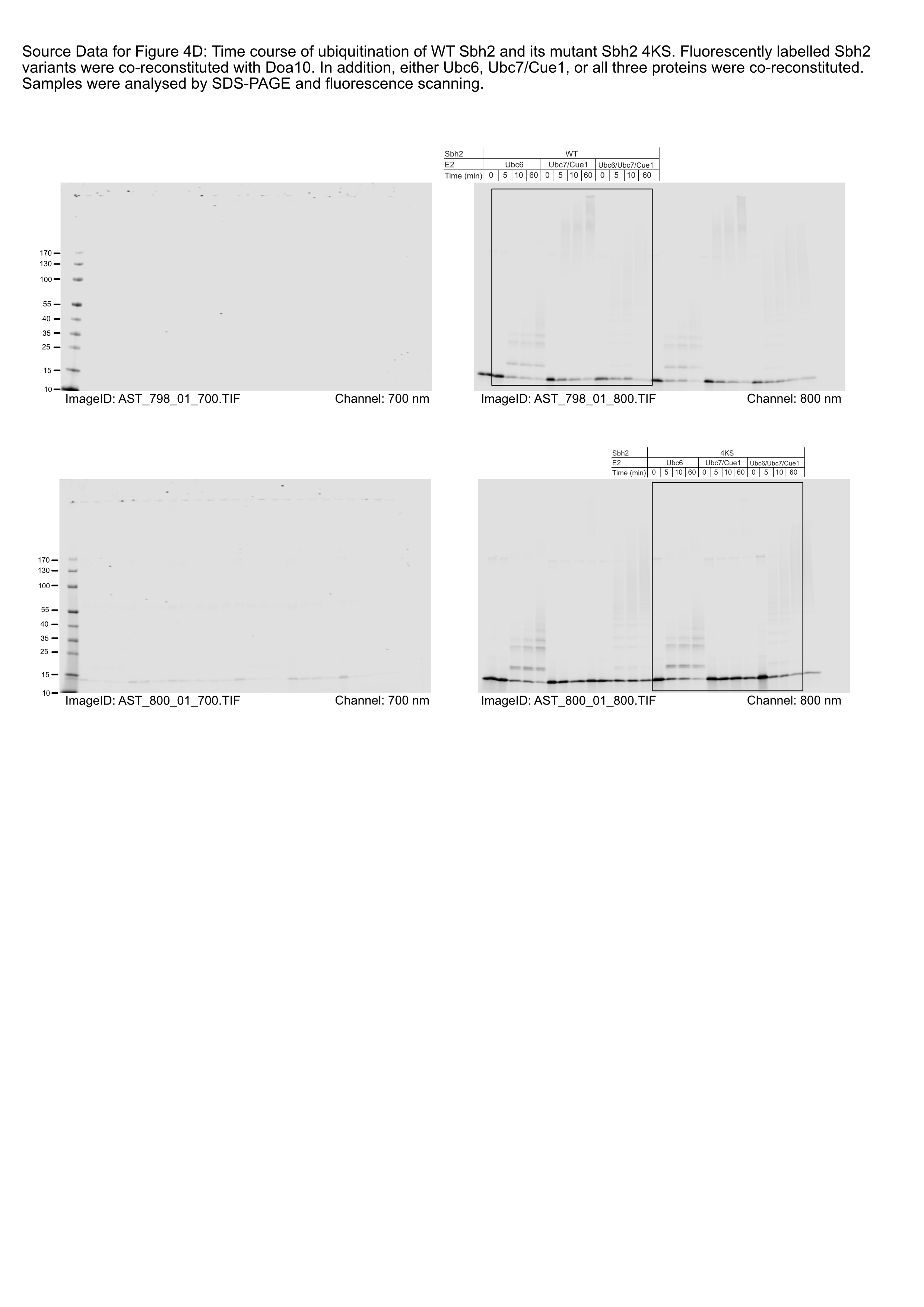

Supplement: Supplementary file 8 — Source data Fig. 4 [file 44318_2024_301_MOESM8_ESM.zip › SD figure 4/SD_Figure_4D.tiff]

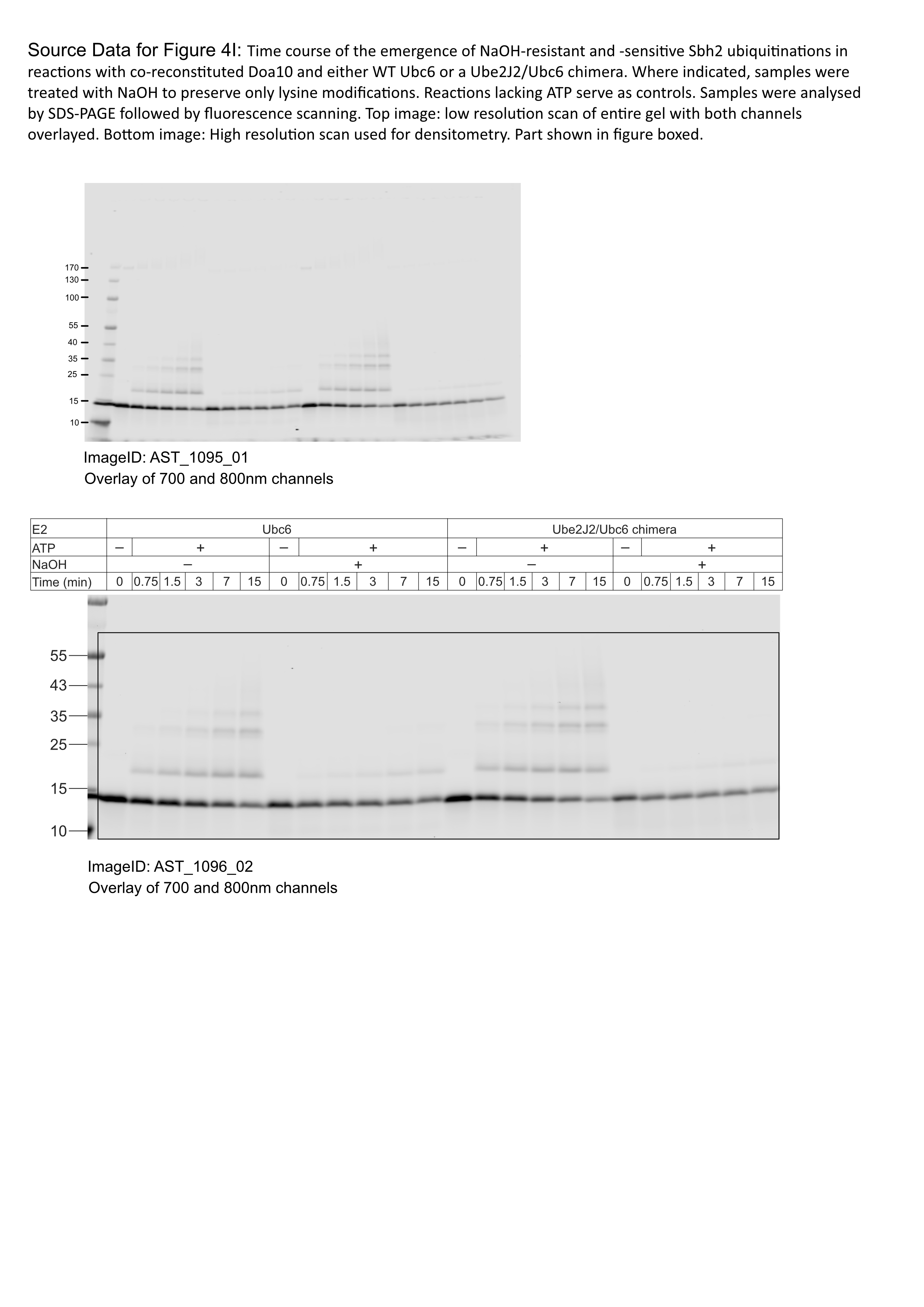

Supplement: Supplementary file 8 — Source data Fig. 4 [file 44318_2024_301_MOESM8_ESM.zip › SD figure 4/SD_Figure_4I.tiff]

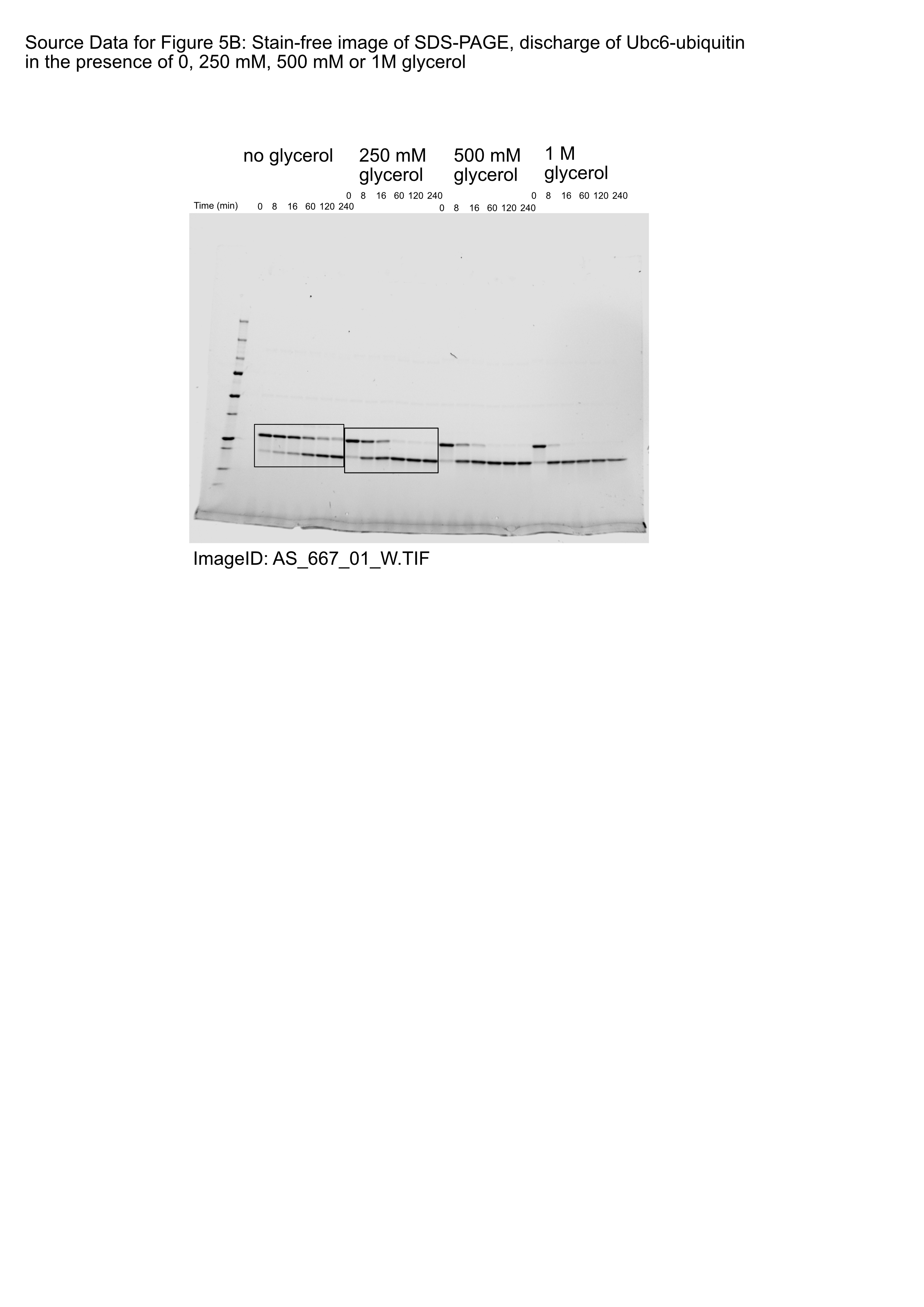

Supplement: Supplementary file 9 — Source data Fig. 5 [file 44318_2024_301_MOESM9_ESM.zip › SD figure 5/SD_Figure_5B.tiff]

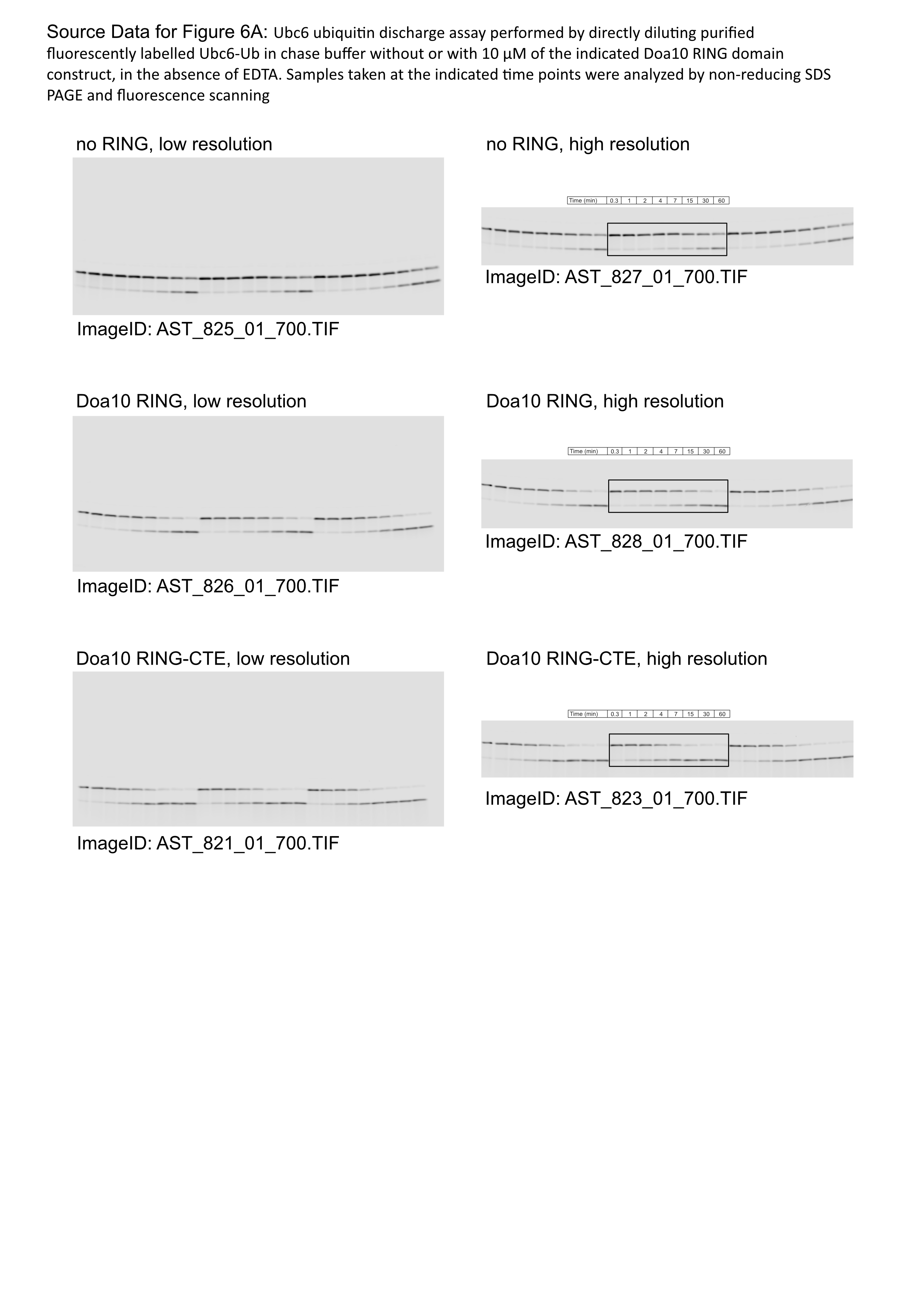

Supplement: Supplementary file 10 — Source data Fig. 6 [file 44318_2024_301_MOESM10_ESM.zip › SD figure 6/SD_Figure_6A.tiff]

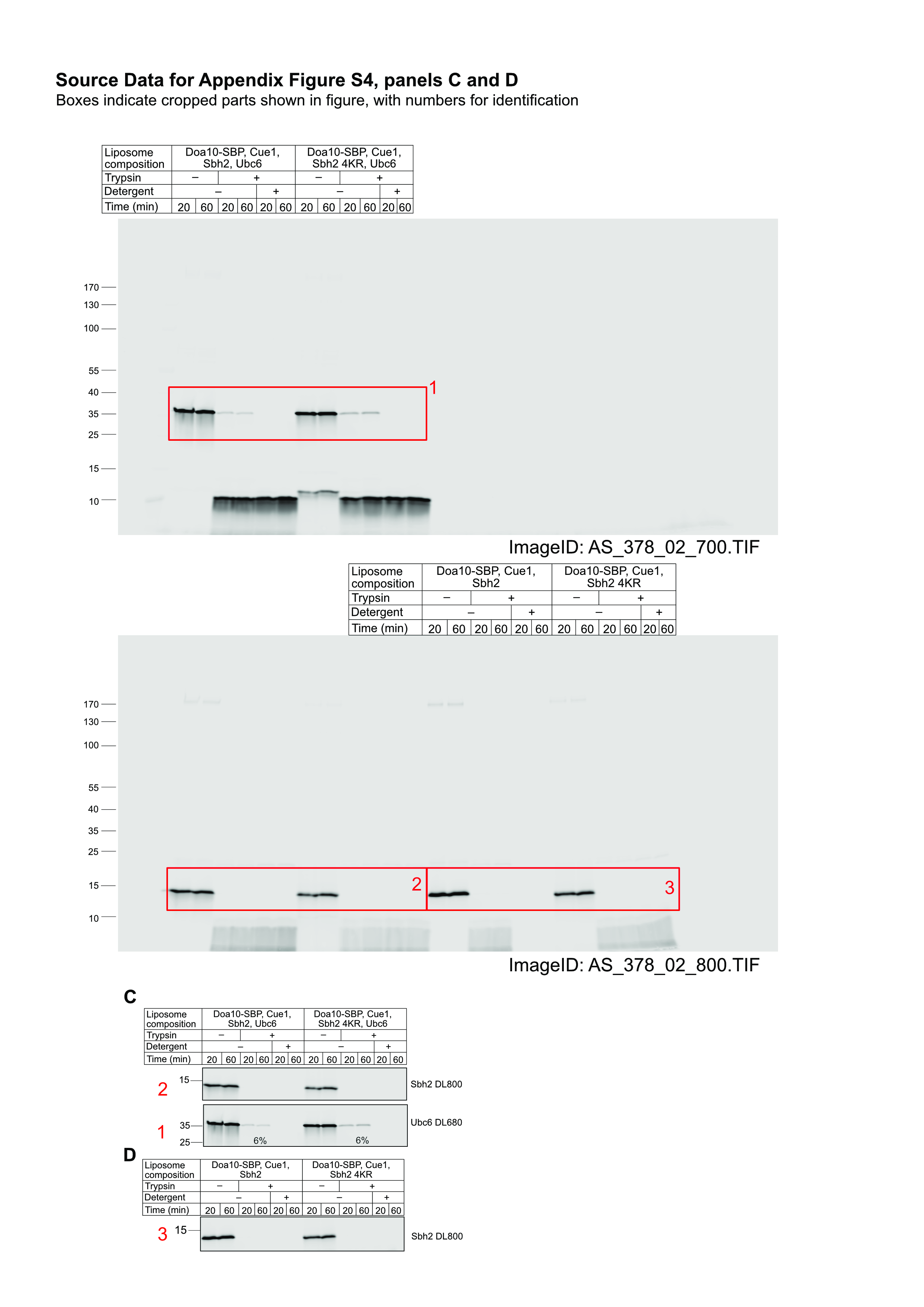

Supplement: Supplementary file 12 — Figure Source Data Appendix S4 [file 44318_2024_301_MOESM12_ESM.zip › Source Data Appendix Figure S4CD/SD_Appendix_Figure_S4CD.tiff]
