# Supplementary material for: Determinants of chemoselectivity in ubiquitination by the J2 family of ubiquitin-conjugating enzymes
Source: EMBO J. 2024 Nov 12;43(24):6705–39. doi: 10.1038/s44318-024-00301-3 (PMC11649903; doi:10.1038/s44318-024-00301-3)
Supplement: Supplementary file 13 — Expanded View Figures [file 44318_2024_301_MOESM13_ESM.pdf]

Expanded View Figures

Figure EV1. Structures and conformational dynamics of Ubc6 and Ubc6-Ub.

(**A**) Electron density map (2Fo-Fc, contoured at the 1.5 σ level) for the active site loop (orange) and the Thr-flap (pink) of the UBC domain of Ubc6 (PDB: 9EN5). (**B**) Comparison of the two copies of the Ubc6-Ub assembly in the asymmetric unit. For alignment in Pymol, the second copy was aligned to the Ubc6 of the first copy. Thr-flap and GRF loop are colored as in Figs. 1 and 2. The active site loop (residues 85–94) is colored orange and red for copies 1 and 2, respectively. The Ubc6-Ub linkage and residue His94 are shown as sticks. The two structures differ markedly in the conformation of the active site proximal region (Lys87-Trp98). Importantly, in the second copy, His94 points away from the ubiquitin attachment site and is involved in crystal packing contacts (not shown). Furthermore, the ubiquitin tails and the relative orientation of ubiquitin towards the UBC domain differ, so that in the second copy, ubiquitin adopts a more "open" conformation (distance $C_{\alpha}Ser113_{Ubc6}$ – $C_{\alpha}Ile44_{Ub}$ 6.8 Å and 9.0 Å for copies 1 and 2, respectively). (**C**) Electron density map (2Fo-Fc, contoured at the 1.0 σ level) for the active site loop (Arg85-His94, in orange) and the ubiquitin tail of the first copy (Leu71-Gly76, in light blue) (PDB: 9EN5). (**D**) The two-dimensional [^{15}N,^{1}H]-HSQC spectrum of ubiquitin. The assignments are indicated by the corresponding number along the protein primary sequence followed by the one-letter amino acid code. (**E**) Plot of rotational correlation time (τ_c) of $Ubc6_{WT}$-(^{15}N)Ub and $Ubc6_{C89A}$-(^{15}N)Ub in blue and red, respectively. For comparison τ_c for free (^{15}N)Ub is shown in gray. Ubc6 and ubiquitin were coupled by a disulfide bond using the ubiquitin mutant G76C. The x-axis denotes residue numbering in ubiquitin.

© The Author(s)

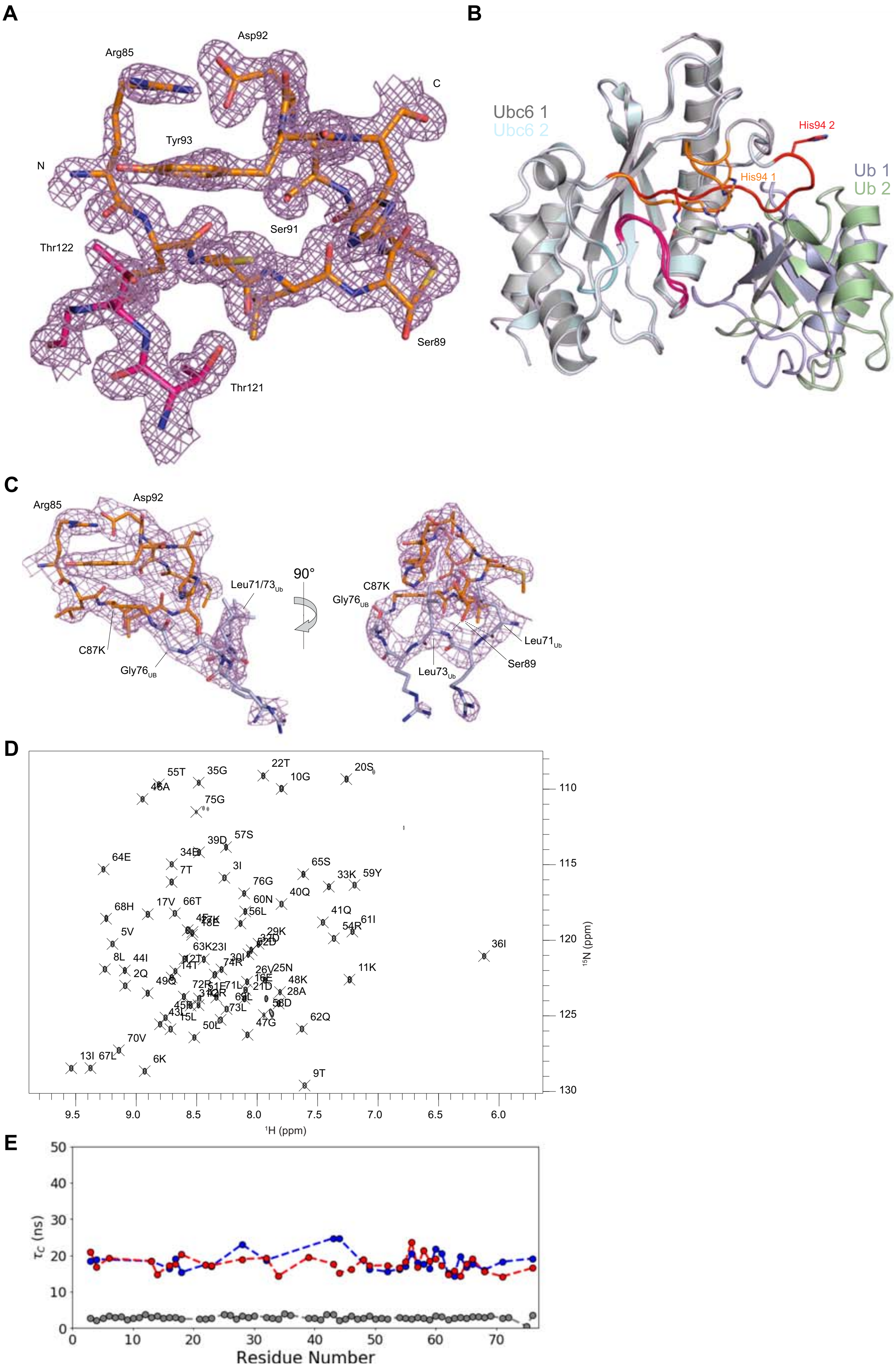
A
Arg85
Asp92
C
Tyr93
N
Ser91
Thr122
Ser89
Thr121
B
Ubc6 1
Ubc6 2
His94 2
His94 1
Ub 1
Ub 2
C
Arg85
Asp92
90°
Leu71/73Ub
C87K
Gly76UB
Leu71Ub
Ser89
Leu73Ub
D
1H (ppm)
15N (ppm)
E
τC (ns)
Residue Number

 © The Author(s)

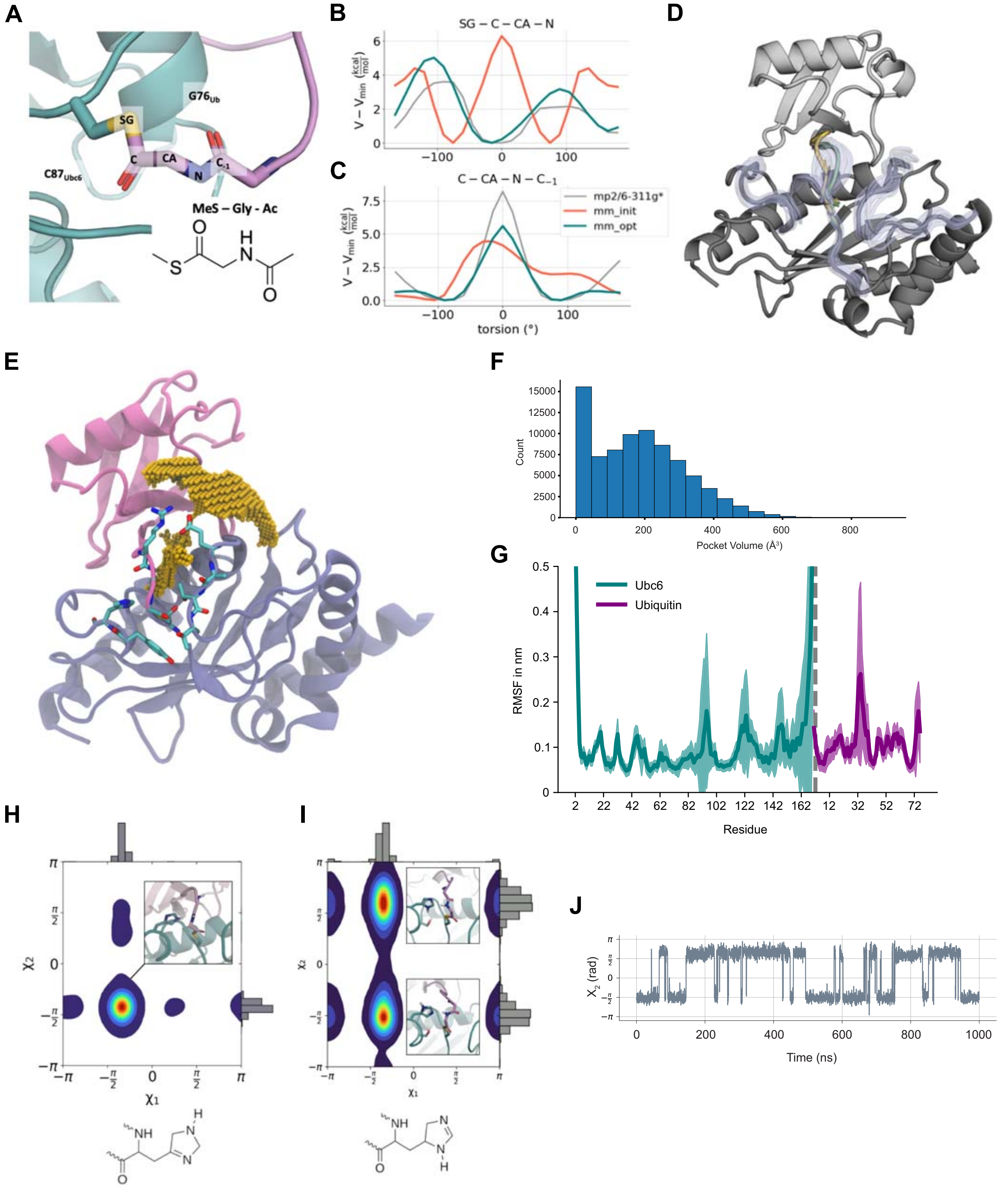
A
G76Ub
SG
C87Ubc6
C
CA
N
C-1
MeS – Gly - Ac
B
SG – C – CA – N
V – Vmin (kcal/mol)
C
C – CA – N – C-1
mp2/6-311g*
mm_init
mm_opt
torsion (°)
D
E
F
Count
Pocket Volume (Å3)
G
Ubc6
Ubiquitin
RMSF in nm
Residue
H
χ2
χ1
I
J
X2 (rad)
Time (ns)

Figure EV2. Molecular dynamics simulations of Ubc6-Ub.

(**A**) Structure of the $C87_{Ubc6}$-$G76_{Ub}$ thioester bond. The residues involved are shown as sticks, the rest of the complex is shown in cartoon representation. The inset shows the capped glycine used for assignment and parameterization of the force field parameters. (**B**, **C**) Optimization of force field parameters. (**B**) Dihedral scans of the SG-C-CA-N (Ψ) torsion. QM potential is shown in gray, the initial force field based potential is shown in red and the optimized potential is shown in teal. All potentials were zeroed on the minimum. (**C**) Dihedral scans of the C-CA-N-C_{-1} (ϕ) torsion. Plot structure is the same as (**B**). (**D**) Cartoon representation of the average structure of the native Ubc6-Ub complex obtained by the simulated annealing procedure. Ubc6 is colored in dark gray, ubiquitin in light gray. The isopeptide-linked Ub-tail from the crystal structure is depicted in yellow, the thioester-linked tail in green. For the parts of the model that were not restrained during simulated annealing (Ubc6 residues 79-98 and 115-128; ubiquitin residues 71-76), every tenth configuration is overlaid in transparent blue. (**E**) A solvent-filled cavity is present near the active site of the Ubc6-Ub complex. Key residues involved in the formation of this cavity are represented as cyan sticks; the rest of Ubc6 and ubiquitin colored in blue and pink, respectively. Small yellow spheres outline the pocket volume, averaged over all configurations generated during simulated annealing. (**F**) Volume of the solvent-filled cavity enclosed by the C-terminal tail of ubiquitin. The average volume was 304 s.d. 219 $Å^3$. (**G**) Root mean square fluctuation (RMSF) of C_α atoms of the Ubc6 (teal) - ubiquitin (purple) complex. The solid line indicates the average of 100 × 1 µs simulations, the shaded area indicates the mean RMSF ± 2σ. (**H**) Contour plot showing the distribution of the χ_1 (N-C_α-C_β-C_γ) and χ_2 (C_α-C_β-C_γ-N_δ) dihedral angles of His94 with the ε-nitrogen protonated. Each contour level corresponds to 10% of the probability density. The inset plot shows the major configuration (χ_1: -64° χ_2: -84°). The ε-protonated nitrogen appears to be incompatible with His94 acting as a base, because its imidazole ring was largely confined to a conformation in which the free electron pair of the δ-nitrogen points away from the region from which a nucleophilic attack would occur. For visualization purposes, 200,000 random samples of the χ1 and χ2 values were calculated from the simulation data. Thereafter each sample was incremented by a value chosen at random from [-2π, 0, 2π]; this abrogated the hard boundaries at -π and π. The density was estimated from the resampled data using a Gaussian kernel density estimator. (**I**) Contour plot showing the distribution of the χ_1 and χ_2 dihedral angles of His94 with the δ-nitrogen protonated. Each contour level corresponds to 10% of the probability density. The inset plots depict the two major conformations (χ_1: -64° χ_2: -88°) and (χ_1: -63° χ_2: 109°), that appeared with similar frequencies. 52% of the structures adopted an N_ε outward-facing orientation, 40% adopted the inward-facing orientation, compatible with a base function for His94. An additional conformation, accounting for 8% of the observed structures, exhibited a χ_1 dihedral angle rotated by 180°. The histograms at the margins show the distributions of each dihedral angle. The dihedral angles were resampled as described in (**H**). (**J**) Exemplary time trace of the χ_2 dihedral angles from simulations of the HisD tautomer. The inward and outward-facing conformations interconverted rapidly during simulations and are only separated by an energy barrier of approximately 1 k_BT.

 © The Author(s)

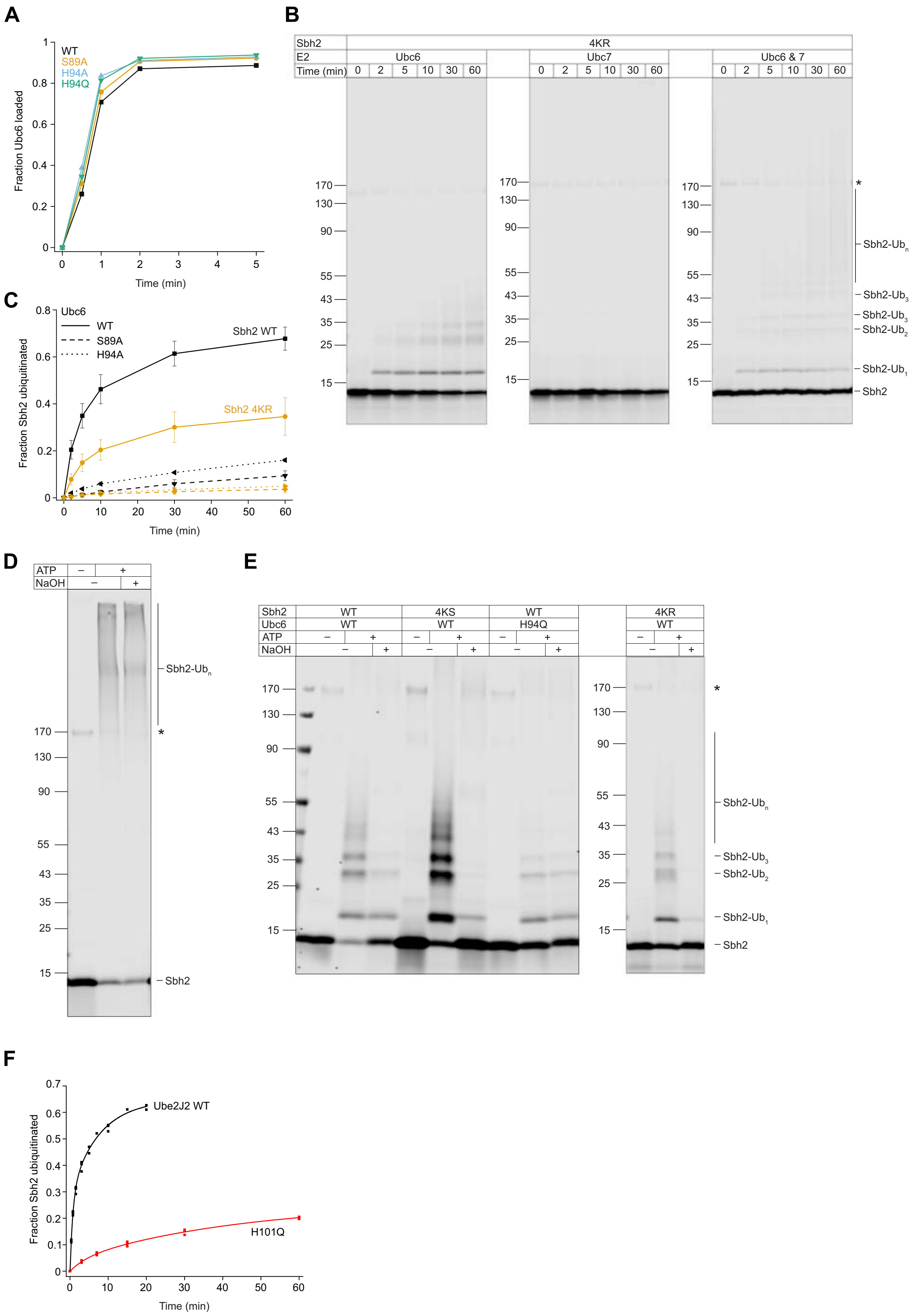
A
B
C
D
E
F
Fraction Ubc6 loaded
Time (min)
WT
S89A
H94A
H94Q
Sbh2
E2
Time (min)
4KR
Ubc6
Ubc7
Ubc6 & 7
Sbh2-Ub$_n$
Sbh2-Ub$_3$
Sbh2-Ub$_2$
Sbh2-Ub$_1$
Sbh2
Fraction Sbh2 ubiquitinated
Ubc6
Sbh2 WT
Sbh2 4KR
ATP
NaOH
4KS
H94Q
Ube2J2 WT
H101Q

Figure EV3. Reconstitution of Sbh2 ubiquitination.

(**A**) E1-mediated Ub loading of full-length WT Ubc6 is unaffected in the indicated mutants. Detergent solubilized WT Ubc6 or the indicated mutants were incubated with E1, Ub and ATP. Time course of E1-mediated loading of fluorescently labeled WT Ubc6 and mutants were analyzed by non-reducing SDS-PAGE and detected by fluorescence scanning. Values for each time point are shown as colored symbols connected by solid lines. (**B**) Time course of ubiquitination of Sbh2 4KR. Fluorescently labeled Sbh2 4KR was co-reconstituted with Doa10. In addition, the indicated E2s/cofactors were co-reconstituted. Samples were analyzed by SDS-PAGE and fluorescence scanning. The asterisk marks a band that appears upon co-reconstitution of Doa10 with fluorescently labeled Sbh2. It probably reflects a partially SDS-resistant complex of the two proteins. (**C**) Quantification of Sbh2 and Sbh2 4KR ubiquitination in the presence of Doa10 and either WT Ubc6 or Ubc6 mutants S89A and H94A. Data points and error bars indicate mean ± one standard deviation from three experiments. Black for WT Sbh2, yellow for Sbh2 4KR. Solid, dashed and dotted lines for WT Ubc6, Ubc6 S89A, and Ubc6 H94A, respectively. Data for WT Ubc6 is reproduced here from Fig. 4E. (**D**, **E**) Representative SDS-PAGE for determining NaOH-resistant and -sensitive Sbh2 ubiquitinations from reactions with either WT Sbh2, Sbh2 4KR, or Sbh2 4KS, co-reconstituted with Doa10 and either Ubc7/Cue1 (**D**) or the indicated Ubc6 version (**E**). Samples were collected after 1 h from reactions as in Fig. 4D. Where indicated, samples were treated with NaOH to preserve only lysine modifications. Reactions lacking ATP serve as controls. Samples were analyzed by SDS-PAGE and fluorescence scanning. As in (**B**), the asterisk marks a band that appears upon co-reconstitution of Doa10 with fluorescently labeled Sbh2. (**F**) Time course of Sbh2 ubiquitination in liposomes containing Sbh2, Doa10 and the H101Q mutant of the Ube2J2/Ubc6 chimera. Data for the non-mutated chimera is reproduced from Fig. 4J for comparison. For visualization, double-exponential fits to the data are shown as solid lines. $N = 3$.

 © The Author(s)

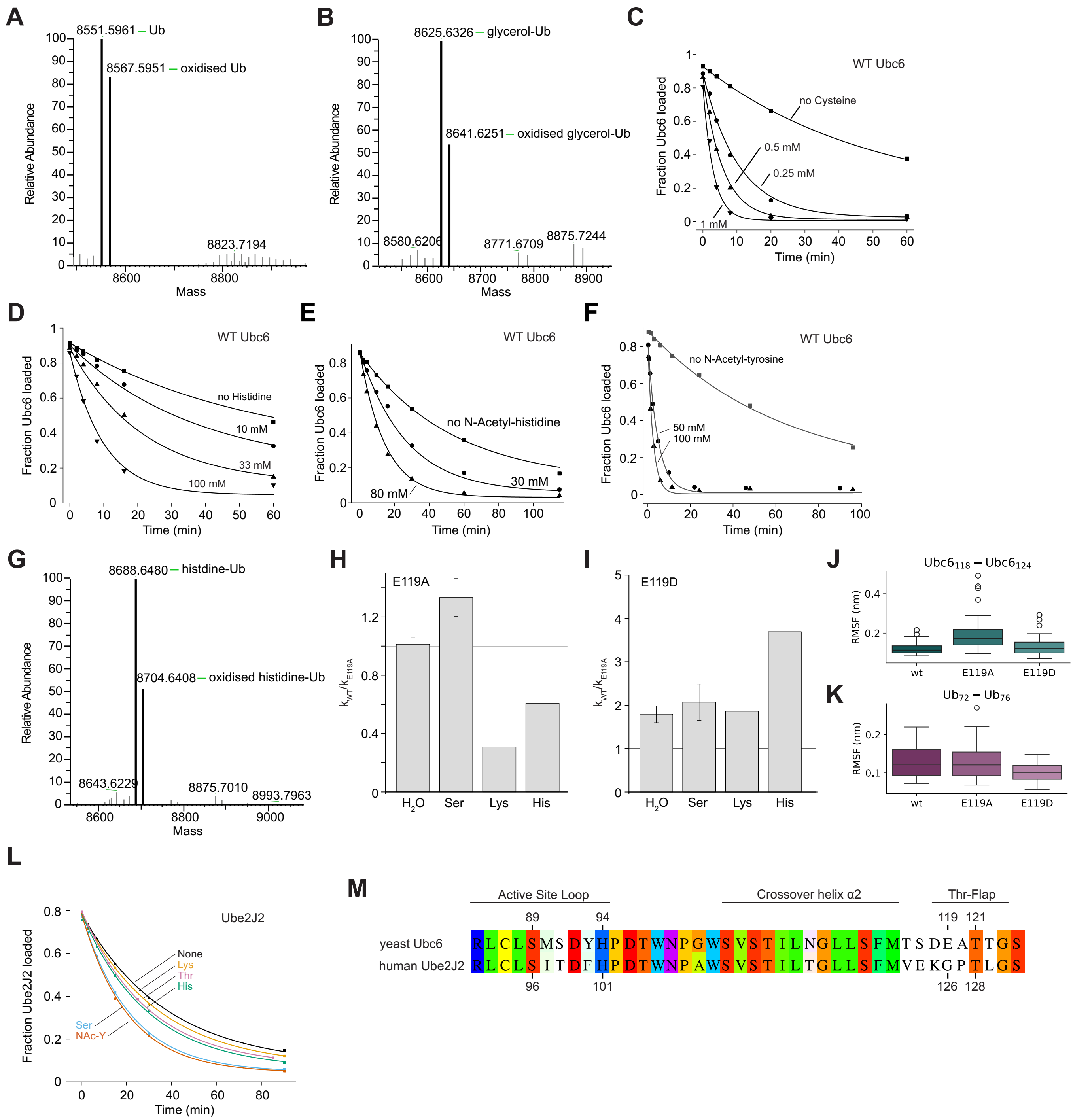
A
8551.5961 – Ub
8567.5951 – oxidised Ub
8823.7194
Relative Abundance
Mass
B
8625.6326 – glycerol-Ub
8641.6251 – oxidised glycerol-Ub
8580.6206
8771.6709
8875.7244
Relative Abundance
Mass
C
WT Ubc6
no Cysteine
0.5 mM
0.25 mM
1 mM
Fraction Ubc6 loaded
Time (min)
D
WT Ubc6
no Histidine
10 mM
33 mM
100 mM
Fraction Ubc6 loaded
Time (min)
E
WT Ubc6
no N-Acetyl-histidine
30 mM
80 mM
Fraction Ubc6 loaded
Time (min)
F
WT Ubc6
no N-Acetyl-tyrosine
50 mM
100 mM
Fraction Ubc6 loaded
Time (min)
G
8688.6480 – histidine-Ub
8704.6408 – oxidised histidine-Ub
8643.6229
8875.7010
8993.7963
Relative Abundance
Mass
H
E119A
k_{WT}/k_{E119A}
H2O
Ser
Lys
His
I
E119D
k_{WT}/k_{E119A}
H2O
Ser
Lys
His
J
$Ubc6_{118} - Ubc6_{124}$
RMSF (nm)
wt
E119A
E119D
K
$Ub_{72} - Ub_{76}$
RMSF (nm)
wt
E119A
E119D
L
Ube2J2
None
Lys
Thr
His
Ser
NAc-Y
Fraction Ube2J2 loaded
Time (min)
M
Active Site Loop
Crossover helix α2
Thr-Flap
89
94
119
121
yeast Ubc6
R L C L S M S D Y H P D T W N P G W S V S T I L N G L L S F M T S D E A T T G S
human Ube2J2
R L C L S I T D F H P D T W N P A W S V S T I L T G L L S F M V E K G P T L G S
96
101
126
128

Figure EV4. Discharge of Ubc6-Ub and J2-Ub by small nucleophiles.

(**A**, **B**) Deconvoluted mass spectra of yeast ubiquitin (**A**) (mass 8551.5960 Da, reference 8551.5747 Da) and glycerol-ubiquitin (**B**) (mass 8625.6326 Da, reference 8625.6115 Da). The mass difference of 74 Da corresponds to the mass of glycerol minus one water molecule. (**C–F**) Ubiquitin discharge assays of WT Ubc6 in the presence of the indicated concentrations of free amino acids. (**C**) Cysteine, (**D**) histidine, (**E**) N-acetyl-histidine, (**F**) N-acetyl-tyrosine (NAc-Tyr). The fraction of the loaded and discharged state were quantified by densitometry. In each panel, the data were globally fitted to determine rate constants for hydrolysis, and discharge by the indicated free amino acid. Fitting results are shown solid lines; the obtained values for histidine and NAc-Tyr are reported in Fig. 5E. (**G**) Deconvoluted mass spectrum of histidine-ubiquitin (mass 8688.6482 Da, reference 8688.6336 Da). (**H**, **I**) Bar plot comparing reactivity of the Ubc6 mutant E119A (**H**) or E119D (**I**) with WT Ubc6 towards the indicated nucleophiles. Discharge by hydrolysis is indicated as "H_2O". Rate constants of mutants for different nucleophiles were determined as in Fig. 5D and averages determined as described for Fig. 5E. Plotted are fold differences of WT/mutant discharge rates. N for the E119A mutant towards the nucleophiles "H_2O", serine, lysine, and histidine is three, two, one, and one, respectively. For the E119D it is three, three, one, and one, respectively. Error bars represent standards deviations as described for Fig. 5F–I. As only single measurements were performed in the presence of free histidine or lysine, these values have no error bars. (**J**) Boxplot comparing the root mean square fluctuation (RMSF) of the $Ubc6_{118}$ - $Ubc6_{124}$ loop between WT Ubc6 and the E119A and E119D mutants. The median and interquartile range between the 25th and 75th percentiles is shown by the boxed area. The center line shows the median. The whiskers extend to all data points within 1.5 times the interquartile range, while all additional points beyond this range are shown as dots. For each variant, 8 simulations were used to calculate the RMSF values of the 7 residues ($n = 56$). (**K**) Boxplot comparing the RMSF of the ubiquitin tail (residues 72-76) between WT Ubc6 and the mutants E119A and E119D. The structure of the boxplots is identical to (**J**). For each variant, 8 simulations were used to calculate the RMSF values of the 5 residues ($n = 40$). (**L**) Ubiquitin discharge assay with the UBC domain of human Ube2J2 in the presence of 50 mM of the indicated nucleophiles. Samples taken at the indicated time points were analyzed by non-reducing SDS-PAGE and stain-free imaging. Plots of fractions of Ub-loaded Ube2J2 were globally fitted. Fitting results are shown as solid lines and values are reported in Fig. 5M. (**M**) Pair-wise alignment of yeast Ubc6 and human Ube2J2 in the region covering the active site loop and the Thr-flap. Source data are available online for this figure.

 © The Author(s)

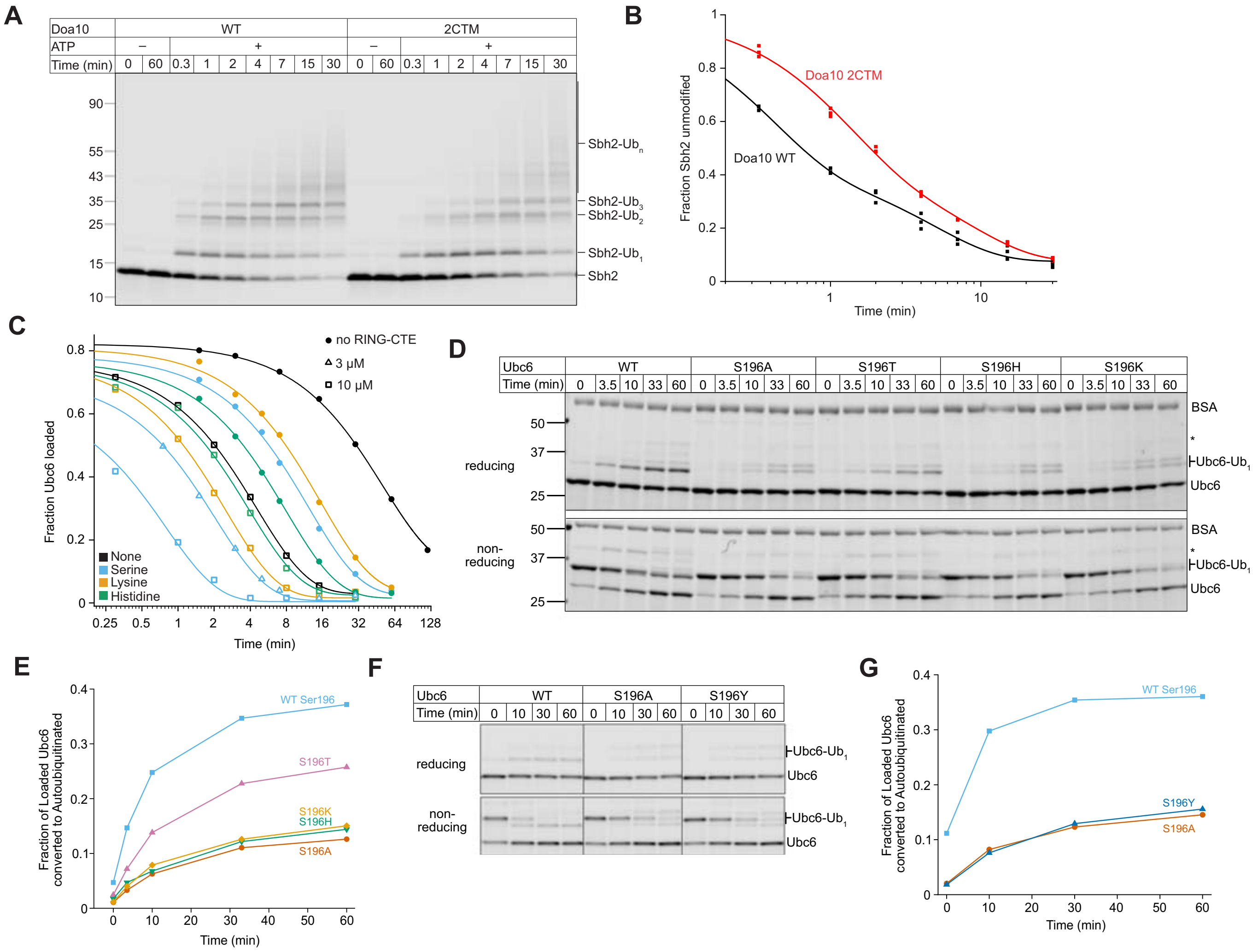

Figure EV5. RING-effect on Ubc6 and Ube2J2 activity.

(**A**) Full-length WT Doa10 is more reactive in Sbh2 ubiquitination than its CTE mutant. Fluorescently labeled Sbh2 was co-reconstituted with Ubc6 and either WT Doa10 or the Doa10 2CTM mutant (G1308L, N1314A). Proteoliposomes were incubated with 1 μM Uba1, 120 μM ubiquitin, and ATP. Samples taken at the indicated time points were analyzed by SDS-PAGE and fluorescence scanning. Indicated samples lacked ATP as a negative control. (**B**) Quantification of the fraction of unmodified Sbh2 from three experiments as in (A). Solid lines represent global double-exponential fits of the data. (**C**) Ubiquitin discharge assays performed with WT UBC domain of Ubc6 in the presence of 50 mM of the indicated free amino acids and the indicated Doa10 RING-CTE concentrations. Plots of the fraction of loaded Ubc6 were globally fitted to a mono-exponential function to determine apparent rate constants for hydrolysis and discharge by free amino acids. Solid lines represent fit results. Such data was used to generate the plot in Fig. 6D. (**D**) Autoubiquitination assay comparing full-length WT Ubc6 and its indicated Ser196 point mutants. A discharge assay with full-length Ubc6 was performed in the presence of the detergent n-dodecyl-β-D-maltoside. After the loading step, EDTA was added to quench E1 activity. Samples collected at indicated time points were analyzed by reducing (top) and non-reducing (bottom) SDS-PAGE and stain-free imaging. The asterisk indicates a band that probably arises from a small fraction of Ubc6-Ub that is autoubiquitinated and Ub-loaded, and that partially converts into a double autoubiquitinated species. (**E**) Quantification of (**D**). The fraction of autoubiquitinated Ubc6 was determined by densitometry from the reducing gel and normalized to the loaded fraction at $t = 0$, as determined from the non-reducing gel. (**F**) As in (**D**), but using the indicated fluorescently labeled Ubc6 variants. Samples were analyzed by SDS-PAGE and fluorescence scanning. (**G**) Quantification of (**F**), as described in (**E**).

© The Author(s)
